# Light–Material Interactions Using Laser and Flash Sources for Energy Conversion and Storage Applications

**DOI:** 10.1007/s40820-024-01483-5

**Published:** 2024-08-26

**Authors:** Jung Hwan Park, Srinivas Pattipaka, Geon-Tae Hwang, Minok Park, Yu Mi Woo, Young Bin Kim, Han Eol Lee, Chang Kyu Jeong, Tiandong Zhang, Yuho Min, Kwi-Il Park, Keon Jae Lee, Jungho Ryu

**Affiliations:** 1 Department of Mechanical Engineering (Department of Aeronautics, Mechanical and Electronic Convergence Engineering), Kumoh National Institute of Technology, 61, Daehak-Ro, Gumi, Gyeongbuk 39177 Republic of Korea; 2 Department of Materials Science and Engineering, Pukyong National University, 45, Yongso-Ro, Nam-Gu, Busan, 48513 Republic of Korea; 3 Energy Technologies Area, Lawrence Berkeley National Laboratory, Berkeley, CA 94720 USA; 4 Department of Materials Science and Engineering, Korea Advanced Institute of Science and Technology (KAIST), 291 Daehak-Ro, Yuseong-Gu, Daejeon, 34141 Republic of Korea; 5 Division of Advanced Materials Engineering, Jeonbuk National University, Jeonju, 54896 Jeonbuk Republic of Korea; 6 School of Electrical and Electronic Engineering, Harbin University of Science and Technology, Harbin, 150080 People’s Republic of China; 7 Key Laboratory of Engineering Dielectrics and Its Application, Ministry of Education, Harbin University of Science and Technology, Harbin, 150080 People’s Republic of China; 8 Department of Materials Science and Metallurgical Engineering, Kyungpook National University, 80 Daehak-Ro, Buk-Gu, Daegu, 41566 Republic of Korea; 9 School of Materials Science and Engineering, Yeungnam University, Daehak-Ro, Gyeongsan-Si, 38541 Gyeongsangbuk-do Republic of Korea

**Keywords:** Light, Light–material interaction, Nanomaterials, Energy conversion and storage devices

## Abstract

This review provides a comprehensive overview of the progress in light–material interactions (LMIs), focusing on lasers and flash lights for energy conversion and storage applications. We discuss intricate LMI parameters such as light sources, interaction time, and fluence to elucidate their importance in material processing. In addition, this study covers various light-induced photothermal and photochemical processes ranging from melting, crystallization, and ablation to doping and synthesis, which are essential for developing energy materials and devices. Finally, we present extensive energy conversion and storage applications demonstrated by LMI technologies, including energy harvesters, sensors, capacitors, and batteries. Despite the several challenges associated with LMIs, such as complex mechanisms, and high-degrees of freedom, we believe that substantial contributions and potential for the commercialization of future energy systems can be achieved by advancing optical technologies through comprehensive academic research and multidisciplinary collaborations.

## Highlights

- This review paper provides a comprehensive analysis of light–material interaction (LMI) parameters, offering insights into their significance in material processing.
- It examines a wide array of photothermal and photochemical processes, showcasing their versatility in creating advanced materials for energy conversion and storage applications.
- The review presents a multidisciplinary approach to advancing LMI technologies and highlights their potential contribution to the commercialization of future energy conversion and storage systems.

## Graphical abstract

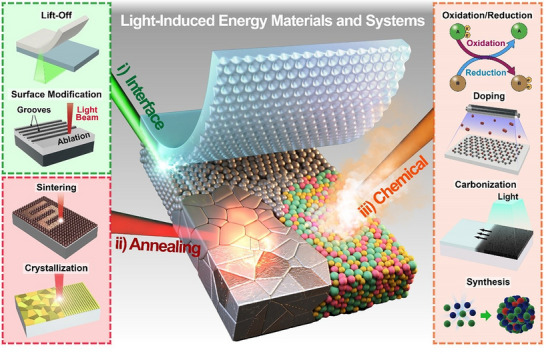

## Introduction

Significant advancements in energy device technologies have profoundly impacted our daily lives by revolutionizing the management, consumption, storage, and generation of energy. These innovations provide effective solutions to the increasing energy demands, fostering clean energy, sustainable communities, and enhanced overall well-being [1–10]. For example, advancements in energy storage systems (ESSs) have led to the proliferation of portable electronics such as smartphones, laptops, wearable sensors, and Internet of Things (IoTs). These technological improvements have developed various societal sectors, such as communication, data collection, automation, and entertainment, enabling individuals to stay connected, productive, and efficient [11–14]. ESSs are expanding to various energy conversion applications, such as solar cells, energy harvesters, and optoelectronics for realizing renewable energy, biomedical healthcare, and self-powered electronic systems [15–31].

Conventionally, thermal treatment of the functional energy materials such as electro-ceramics, metal oxides, silicon, carbon materials, and perovskites is performed in a furnace at high temperatures of 1000 °C or above, depending on the material systems and the intended microstructures. However, the furnace annealing method requires long processing times and a large amount of energy, because most all of the energy is used to gradually raise and lower the system temperature, including the target sample and furnace. These features impede achieving rapid and controlled thermal treatment of energy materials to minimize undesirable effects such as severe oxidation, microstructural damages, thermal expansion mismatch, and mechanical failures. In addition, energy materials must be physically separated from the electronic circuitry during furnace heating, limiting the on-chip integration of energy applications.

Light–material interaction (LMI) processes have emerged as promising candidates for investigating energy devices owing to their exclusive capability to induce instantaneous, multiphysical, spatiotemporally controlled, nonequilibrium photon reactions, which are difficult under traditional microfabrication and thermal processes [32–38]. Among numerous light sources, including light-emitting diodes (LEDs), sunlight, lasers and flash lamps have been extensively employed because of their capability to irradiate high-intensity photon energy, as presented in Table 1. Lasers operate based on light amplification by the stimulated emission of radiation, which enables the emission of high-power photon energy. The intense laser beam can be further focused in an extremely localized area through optics owing to its excellent directionality and coherence. Flash lamps irradiate high-intensity pulsed light through intense electrical discharging, which can rapidly increase the temperature of energy materials within milliseconds by delivering substantial energy to the processed material. However, wavelength characteristics of the light emitted by lasers and flash lamps are different, which leads to distinctive LMI tendency. Laser sources generate monochromatic light of a specific wavelength, enabling a high-absorption efficiency by selecting a wavelength suitable for target energy materials. Flash lamps produce a broad light spectrum, ranging from ultraviolet to infrared. This wide range of wavelengths allows materials (with diverse compositions and arrangements) to absorb appropriate photon energies and induce effective annealing to improve the material properties of energy device components. For example, the broad flash light spectrum can excite multiple plasmonic modes of randomly arranged nanowires (NWs), resulting in a uniform plasmonic welding effect [39].

**Table 1:** Comparison of light sources for light-induced energy conversion and storage applications

Types of light sources	Lasers	Flash lamps	LEDs	Sunlight
Capability for high-intensity processing	Very high	High	Low	Very low
Wavelength	Monochromic	Broad spectrum	Narrow band emission	Broad spectrum
Advantages	High controllability	High scalability	Large-area processability	Eco-friendly
	High absorption efficiency	Large-area processability		Abundant in nature
Limitations	Low scalability	Low precision	Limited range of possible light-induced effects for energy applications due to the insufficient light intensity	
	Limited for large-area processing	Limited absorption efficiency		

**Fig. 1:**
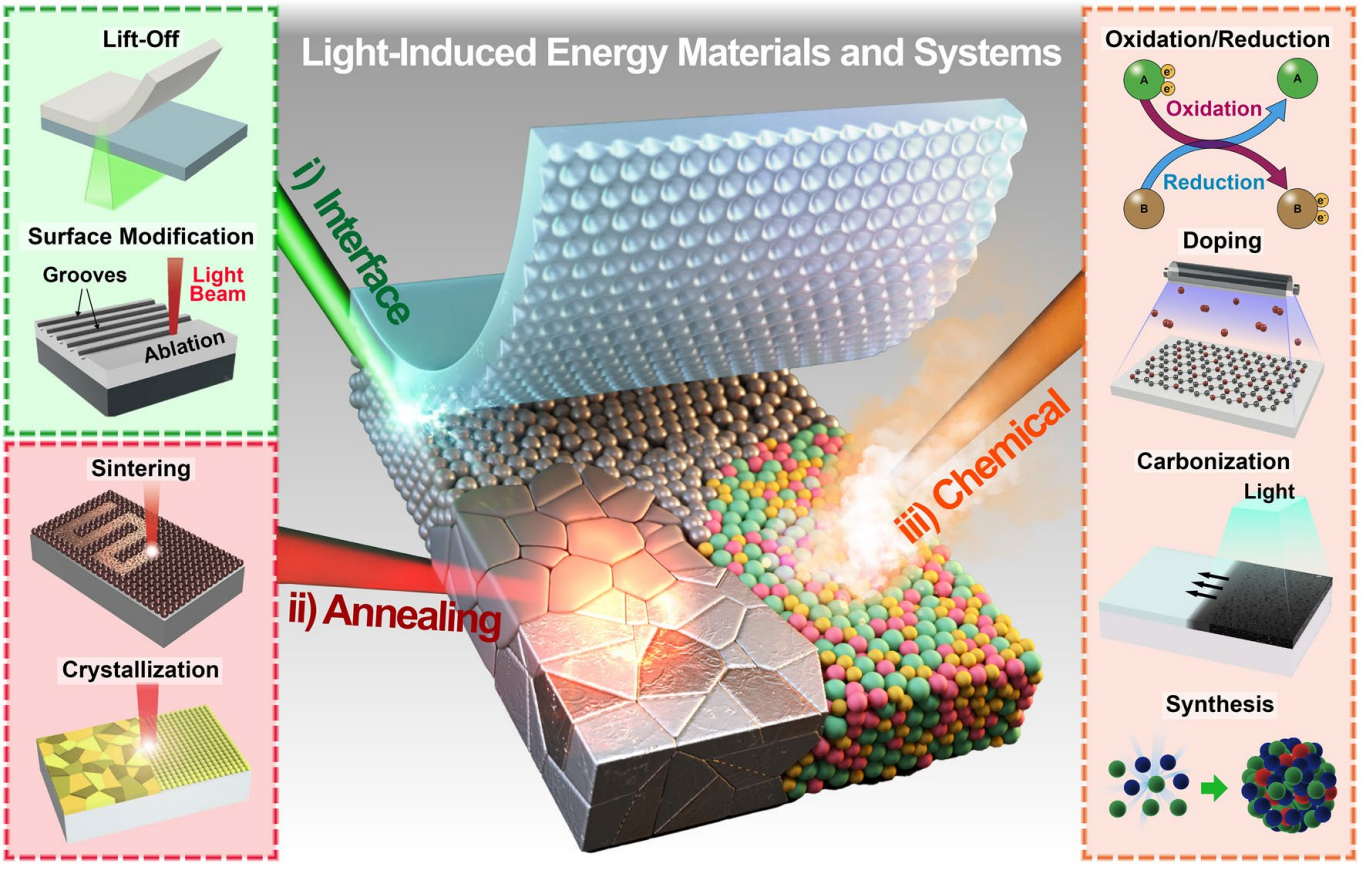
Schematic of the overall concept of light-induced energy materials and devices

Diverse manufacturing techniques have been implemented by lasers and flash lamps, leveraging their respective advantages such as high-precision laser controllability and large-scale flash light processability, respectively, as shown in Fig. 1 [39–46]: (i) High-intensity photon energy directed at the material interface generates extreme temperature gradients within the confined space and time to trigger photothermal reactions, including melting, vaporization, ablation, decomposition, thermal expansion, and delamination. Transient interfacial interactions have been exploited to develop practical approaches, such as lift-off and surface texturing, to transfer the functional layer and maximize the active surface area to improve energy applications [47–58]. (ii) Prolonged optical annealing allows thermal energy to be transferred deep inside the target materials through a heat conduction, leading to numerous volumetric photothermal effects, including sintering, welding, and crystallization [59–62]. Incident photon energy can cause the bonding and assembly of fine materials (from the nano- to the microscale) into a solid by promoting atomic mass transport, thermal diffusion, and fusion between neighboring substances [63–65]. In light-induced melting and solidification processes, materials undergo controlled reordering and realignment of atomic arrangements, transforming amorphous materials into crystalline structures [66, 67]. Photonic sintering and crystallization enhance various energy material properties, including electrical conductivity, electron mobility, dielectric constants, optical absorption, piezoelectricity, and magnetoelectricity [68–70]. (iii) The interactions between light and materials can trigger physical and chemical responses, giving rise to distinctive processes such as oxidation, reduction, doping, dissociation, and synthesis [71–75]. The energy-related properties of photon-reactive materials can be optically modified, improved, and optimized by tailoring their chemical structures to satisfy device performance, functionality, efficiency, and reliability requirements. Recently, these light-derived chemical engineering methods have advanced beyond classical thermodynamics, enabling physicochemical interlocking and synthesis of metastable nanomaterials for creative energy and optoelectronic systems [76, 77].

Here, we provide an overview of the representative progress in light-induced technologies for developing energy materials and systems, elucidating their impact on functionality, performance, and applications. First, we discuss the parameters related to LMI, including light sources, basic principles, and processing factors that affect comprehensive photonic effects in materials. Next, we introduce various photothermal processes, such as sintering, crystallization, and lift-off, as well as photochemical reactions, ranging from doping to synthesis. Finally, a wide spectrum of energy conversion and storage devices demonstrated by LMIs are discussed, including energy harvesters, sensors, capacitors, and batteries.

## Key Processing Parameters for LMIs

**Fig. 2:**
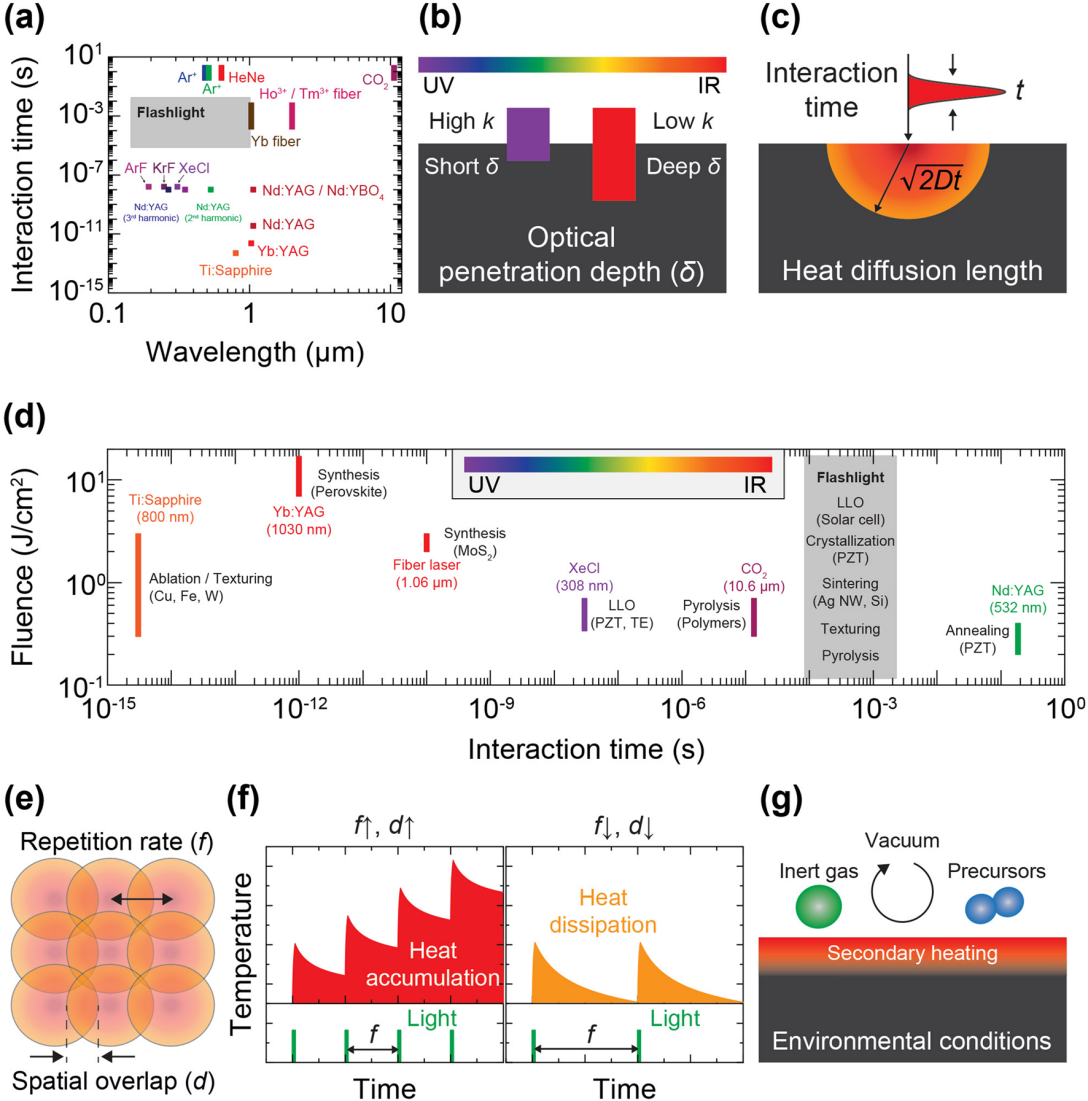
**a** Wavelength and interaction time features of lasers and flash lamps [48, 81–103]; **b** light wavelength influencing the optical penetration depth for light-absorbing materials; **c** interaction time that determines the heat diffusion length; **d** fluence and interaction time regime related to LMI events [81–83, 89, 92–96, 99, 102–104]; **e** repetition rate and spatial overlap, contributing to **f** heat accumulation/dissipation effects; **g** environmental conditions that trigger physicochemical reactions

LMIs can precisely and selectively manipulate thermal energy transport within controlled time intervals, which is challenging to achieve using traditional microfabrication and furnace-based annealing methods. However, achieving the intricate nature of LMIs involves multiple simultaneous parameters that must be considered carefully to achieve the desired physicochemical reactions and their corresponding consequences involves considering multiple simultaneous parameters due to the intricate nature of LMIs. Figure 2 highlights the key factors contributing to the interactions between light and materials, including incident wavelength, irradiation duration, fluence or power, repetition rate, spatial overlap, and environmental conditions.

### Wavelength

Lasers produce monochromatic and coherent radiation of specific wavelengths, which is attributed to the characteristic excitation of the laser medium. In contrast, flash lamps emit photons with a broad optical spectrum ranging from ultraviolet to infrared, as presented in an overview of different lasers and flash lights (Fig. 2a) [70, 78, 79]. For example, a Ti:sapphire femtosecond laser effectively irradiates photons with wavelengths near 800 nm by optically pumping a crystal of sapphire doped with Ti^3+^ ions [80]. In flash lights, the optical spectrum ranging from 190 to 1100 nm is predominantly emitted by ionizing noble gas atoms with quantized energy states through electrical discharging [70]. These wavelengths from the light sources should be carefully selected for LMIs because they are critical in determining efficient light absorption.

The interaction of incident light with light-absorbing materials is characterized by absorption and reflection, which depend on the spectrally varying optical properties of the materials, denoted by the complex refractive indices (*n* + *ik*). Based on the Beer–Lambert law, the light intensity $I$ within the medium and the corresponding optical penetration depth $l$ can be expressed as follows [79]:

1 $$I={I}_{0}{e}^{-\alpha d}, l=\frac{1}{\alpha }=\frac{\lambda }{4\pi k}$$

where ${I}_{0}$ is the incident photon intensity, $\alpha$ is the absorption coefficient, $d$ is the optical path length, $\lambda$ is the light wavelength, and $k$ is the extinction coefficient (imaginary part of the refractive index). A wavelength with a lower absorption coefficient can penetrate deeper into the material than a wavelength with a higher absorption coefficient, as illustrated in Fig. 2b. Near-infrared light generally exhibits a longer optical penetration depth than visible or ultraviolet light for perovskite Pb(Zr_*x*_,Ti_1–*x*_)O_3_ (PZT) and noble metal (e.g., Ag and Au) nanomaterials, allowing photothermal annealing of thick layers by ensuring uniform and volumetric thermal effects across the thickness direction [97, 105, 106]. In contrast, short wavelengths are advantageous in processing materials with a large band gap or thin films (in the order of 100 nm), as well as mitigating thermal damages to heat-vulnerable substrates, such as polyethylene terephthalate (PET), owing to their shallow absorption depth [73, 107, 108].

### Irradiation Time

When lasers or flash lights with distinct pulse durations are irradiated onto the target material surface, various of consecutive LMIs, including prompt absorption of optical energy, photothermal conversion, lattice thermalization, and heat dissipation, occur within a timescale ranging from femtoseconds to milliseconds, which determines the timespan of LMI. The interaction time *t* is typically defined by the characteristic pulse width of pulsed lasers and flash lights. For continuous-wave (CW) lasers, this is determined by the scanning speed and beam spot size [109]. The pulse duration influences the heat diffusion length ${l}_{th}$. This relationship can be expressed as follows:

2 $${l}_{th} \sim \sqrt{2Dt}$$

where $D$ is the thermal diffusivity of the material and $t$ is the interaction time. Therefore, the interaction time is crucial in controlling the heated target material volume (Fig. 2c**)**. Pulsed lasers with short pulse widths are advantageous when high-precision LMIs are desired without causing thermal effects on adjacent materials. For example, femtosecond laser ultrafast pulses can minimize heat-affected zones because the instantaneous energy deposition time is shorter than the electron–phonon relaxation time (in the order of a few picoseconds for metals) [85, 110]. Light pulse durations in the range of 10 µs to 100 ms (thermal flux regime) can be discharged by flash lamps, which facilitates surface annealing of thin films with thickness up to micrometer-scale. In case of CW lasers, optical irradiation time can be further extended by adjusting the laser scanning speed, and focused beam spot size. The prolonged annealing times (in the region of isothermal process) enabled by CW lasers are preferable for volumetric heating effects [70].

Another variable affecting thermal diffusion length is the thermal diffusivity ($D=\frac{k}{\varrho C})$ of the material, which is a function of heat capacity ($C$), density ($\varrho$), and thermal conductivity ($k$) [78, 79]. The thermal diffusion length was estimated to be 31.1 μm for aluminum (*D* ~ 9.7 × 10^–5^ m^2^ s^−1^) and 5.8 μm for stainless steel (*D* ~ 3.3 × 10^–6^ m^2^ s^−1^) under a 1 μs light irradiation because of the significant difference in their thermal conductivities. Hence, LMI interaction time and substrate thermophysical properties must be considered to achieve the desired photothermal processes within a controlled volume.

### Fluence and Intensity

Fluence (J cm^−2^) and intensity (W cm^−2^) are commonly used to quantify the photon energy applied to the material surface. The incident light energy and interaction time establish the surface temperature profiles and heating/cooling rates, leading to diverse LMI phenomena as shown in Fig. 2d. The spatial distribution of the Gaussian laser beam can be expressed, as follows [111]:

3 $$I\left(r,t\right)={I}_{peak}exp(-\frac{{r}^{2}}{{w}^{2}})$$

where, ${I}_{peak}$ is the peak intensity and $w$ is the radius at which the laser intensity drops to 1/e^2^ of ${I}_{peak}$. Therefore, the laser beam has a stronger intensity at the center of the laser (i.e., $r=0$) compared to the peripheral areas. This variation in local laser intensity results in different laser–material interactions, such as hydrodynamic instability, particle generation, and plume formation [112]. Particularly, for pulsed laser beams, the dimensionless temporal profile can be described as follows [111]:

4 $$\text{p}\left(t\right)=\left\{\begin{array}{c}\frac{{I}_{peak}\left(t\right)}{{I}_{peak, max}},\quad  t<{t}_{pulse}\\ 0, \quad  t> {t}_{pulse}\end{array}\right.$$

where ${I}_{peak, max}$ is the maximum peak intensity at $t={t}_{max}$. ${t}_{pulse}$ is typically characterized by the full-width-half-maximum pulse length. Pulsed lasers with unique pulse durations can lead to different LMIs, as discussed in Sect. 2.2.

In the LMI time frame above ~ μs, sufficient light intensity can raise the temperature of materials such as polymers, Ag NWs, oxide ceramics such as PZT, and hybrid perovskites near or beyond their melting point, facilitating different thermodynamic processes, such as sintering, welding, alloying, recrystallization, and pyrolysis [84, 97, 113–115]. These LMIs have been extensively demonstrated using flash lights because of their broad fluence range from 0.1 to 20 J cm^−2^ coupled with LMI time exceeding 100 μs. Laser sources operating with fluence in the range of 0.2–0.7 J cm^−2^ have been applied to initiate localized photothermal responses, such as annealing, pyrolysis, and thermodynamic phase change.

Beyond the ablation threshold, where light fluence is high (> 0.4 J cm^−2^) and interaction time is below 1 μs, vaporization, and plasma formation may occur on the irradiated surfaces. Specifically, the transition of solid material into gaseous phases can selectively remove material from the target surface. Furthermore, recoil pressure applied to the molten liquid pool promotes the ablation process by expelling liquid materials [48]. Ionization of the vapor can lead to strong plasma formation, generating a shock wave. These effects can be strategically leveraged in laser lift-off (LLO), texturing, cutting, drilling, and analytical chemistry [37, 48, 110, 116–118]. For example, an ultrafast femtosecond laser was used to ablate and texture metallic surfaces for solar thermoelectric generation [81]. XeCl excimer lasers with a 30 ns pulse width have been widely used for LLO to transfer pre-assembled devices owing to their ability to melt, dissociate, and vaporize sacrificial materials within tens of nanoseconds [119].

### Repetition Rate and Spatial Overlap

The repetition rate *f* is defined as the number of pulse irradiations per second. The spatial overlap can be determined by factors such as the repetition rate, scanning speed, and spot beam size, as depicted in Fig. 2e [120]. The total energy $P$ deposited at an equivalent location is proportional to the product of the incident pulse energy ${E}_{p}$, repetition rate $f$, and spatial overlap $d$, and is expressed as follows:

5 $$P \sim {E}_{p}\times f \times d$$

Therefore, overlapping LMI material regions can undergo intensified heating at high repetition rates, causing the deposited photon energy to accumulate over time before dissipating into the bulk, as schematically illustrated in Fig. 2f. In contrast, low repetition rates along with narrow spatial overlap allow effective quenching of thermal energy, resulting in uniform LMIs throughout the target sample without detrimental photonic effects in the overlapped region. Strategic manipulation of these two parameters provides control over the heating level, affecting photothermal processes, including sintering, crystallization, welding, texturing, and ablation.

### Environmental Conditions

As illustrated in Fig. 2g, environmental conditions, such as the gas environment and the use of secondary heating methods, can affect LMIs. Diverse processes, including reduction, doping, synthesis, phase separation, and etching, can be initiated depending on the material and background gas conditions (e.g., precursors, inert gases, or vacuum). For example, a 2D transition metal dichalcogenide was synthesized by irradiating a fiber laser (1.06 μm wavelength) in the presence of a precursor [83]. Laser-assisted graphene chlorination was demonstrated using an ultraviolet nanosecond laser, which allowed environmental Cl_2_ gas dissociation and sequential chloride diffusion into graphene [121]. Moreover, different thermal source types, such as a heating stage or dual laser irradiation, can be applied in addition to the primary light source to provide sufficient activation energy for LMIs and control the heating and cooling rates during the light-induced annealing process [100, 107]. Besides heating in gas, other environmental conditions, such as assisting liquid in the form of waterjet, flowing water, chemical solutions, and vacuum, have important influences on the laser machining process. Mohammed et al. [122] synthesized gold nanoparticles by laser ablation in deionized water. Liquid media (chlorine) was utilized as a liquid jet to cut silicon by laser chemical processing and achieved maximum groove depth and form [123]. A high aspect ratio (over 100:1) and high-quality microholes 100 µm diameter were fabricated by femtosecond laser in air and vacuum environments [124]. The laser-induced hydrothermal growth method was demonstrated to grow NWs on a selected area even smaller than the laser focus size by creating a laser absorption layer [125]. This approach enables precise localized temperature control, successfully synthesizing smaller nanowire arrays without complex optics adjustments and can be applied to various nanowires, such as ZnO and TiO_2_, as well as heat-sensitive polymer substrates.

## Examples of LMI for Energy Conversion and Storage Applications

### Lift-Off (Laser + Flash)

The lift-off process has been widely used to transfer entire inorganic thin films or devices onto flexible substrates for use in flexible electronics, optoelectronics, and energy harvesters [23, 51, 89, 103, 116, 126–128]. First, the fabricated electronic devices and thermally annealed inorganic thin films are fixed onto plastic receiver substrates. When a laser beam is directed to the backside of a rigid mother substrate that is transparent to the exposed light wavelength, the irradiated laser passes through the bulk substrate. Subsequently, it reaches the interfacial layer between the active electronics/inorganic thin film and rigid substrate to support photothermal reactions, such as dissociation, explosive gas release, melting, and vaporization. These LMI behaviors enable the thin-layered structure-based devices to be peeled off the rigid substrate without cracking, wrinkling, or mechanical deformation [54, 116, 127]. This capability is important for developing advanced, lightweight, and flexible energy devices that can be integrated into a variety of new applications, such as wearable technology and portable power sources.

**Fig. 3:**
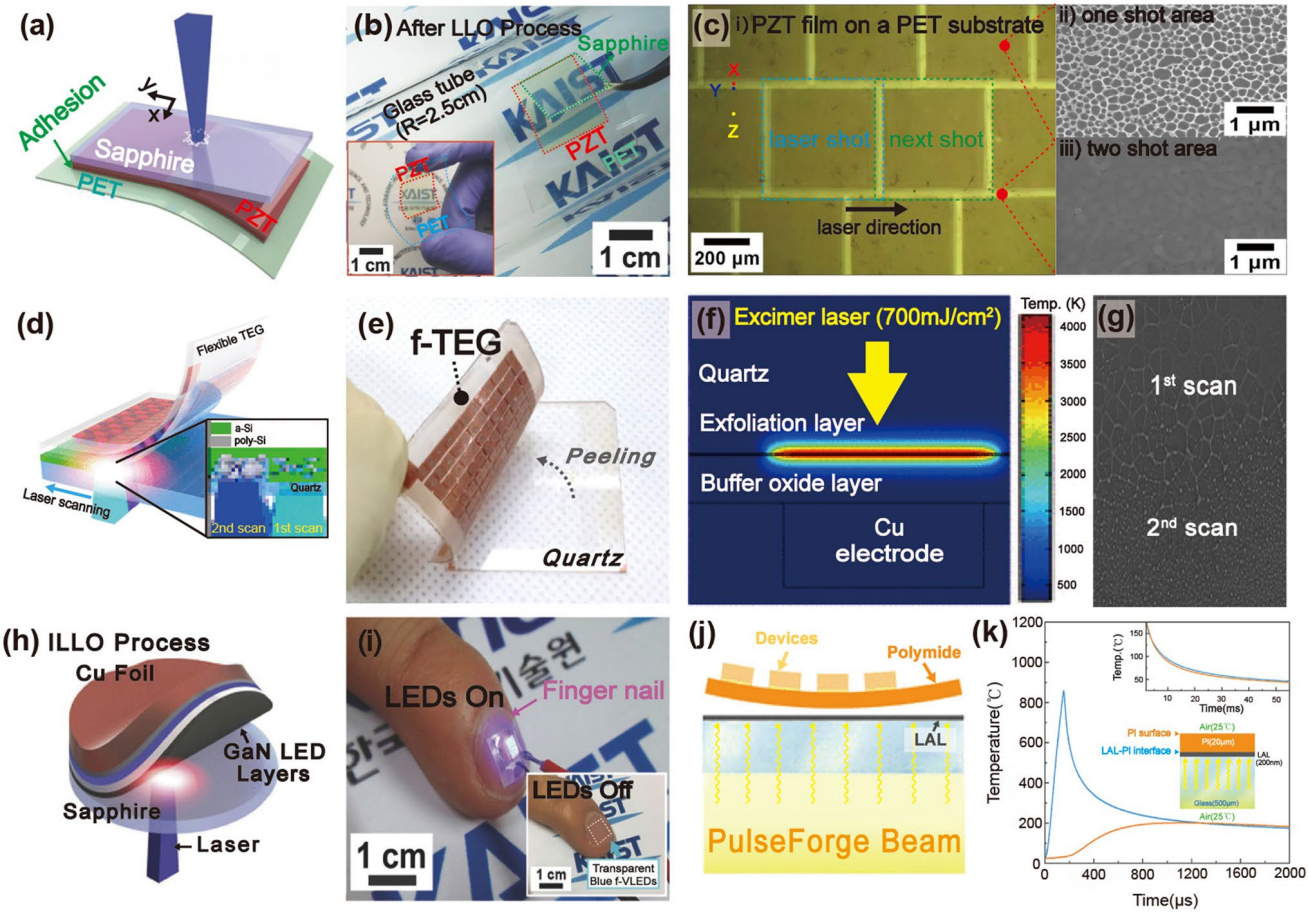
**a** Schematic illustration showing exfoliation step of piezoelectric PZT onto a flexible substrate from a rigid wafer by LLO transfer technique; **b** flexible PZT thin film on plastic substrate is peeled off from a transparent sapphire wafer by laser irradiation; **c** optical image (left panel) and SEM images (right panels) of light source-induced areas of PZT thin film. Reproduced with permission from Ref. [89]. Copyright 2014, Wiley–VCH. **d** Illustration of LLO procedure based on laser multiscanning for fabricating f-TEGs; **e** photograph of f-TEG being exfoliated from bulk quartz; **f** calculated simulation results investigating optimal butter oxide layer thickness using a finite element method; **g** SEM image indicating Si layer exposed areas after laser multiscanning. Reproduced with permission from Ref. [116]. Copyright 2016, American Chemical Society. **h** Fabrication step schematic of KrF laser detaching GaN-based f-VLED from transparent bulk wafer; **i** captured images of transparent, blue-colored f-VLED array transferred onto curved surface of a human fingernail in on and off states. Reproduced with permission from Ref. [127]. Copyright 2018, Wiley–VCH. **j** Illustration showing PV exfoliation from LAL/glass substrate onto PI thin layer using PLO method; **k** simulated temperature change at LAL-PI interface to confirm the feasibility of flexible PV lift-off from a rigid substrate. Reproduced with permission from Ref. [103]. Copyright 2021, American Chemical Society

Park et al. used the LLO process to separate the entire area of a piezoelectric PZT thin film from a transparent sapphire substrate [89]. Figure 3a shows a schematic of the LLO transfer process. The crystallized PZT thin film on the sapphire substrate was transferred onto a flexible PET substrate via laser beam irradiation. Because the XeCl laser photon energy (4.03 eV) is located between the band gap energies (*E*_*g*_) of the sapphire (*E*_*g*_ = 10 eV) and PZT ceramics (*E*_*g*_ = 3.2 ~ 3.6 eV), the penetrating laser beams locally vaporized the laser-absorbing sacrificial layer between the PZT layer and the sapphire substrate, thus delaminating the PZT thin film from the sapphire (see Fig. 3b). As shown in the inset of Fig. 3b, the transferred PZT thin film on a flexible substrate exhibited highly flexible characteristics without cracks and was used to fabricate a large-area, highly efficient, flexible piezoelectric energy harvester. Figure 3c shows optical image (i) and scanning electron microscopy (SEM) images (ii and iii) of the laser-irradiated PZT thin film surface on the PET substrate. To separate the active area from the mother substrate, two-dimensional laser beams with square spots of 500 μm × 500 μm were scanned, overlapping in the x- and y-directions.

Kim et al. reported a high-performance, flexible thermoelectric generator (f-TEG) using the laser multi-scanning (LMS) LLO method [116]. The TEG on a SiO_2_ (buffer oxide layer)/amorphous Si (a-Si, exfoliation layer)/quartz (sacrificial substrate) fabricated via screen printing was immediately peeled off using multiple scans of the XeCl excimer laser, as shown in Fig. 3d, e. Unlike the general LLO process, the laser beam was exposed to the sample several times during LMS LLO to detach the TEG array layers from the quartz substrate by reducing adhesion between the a-Si and sacrificial quartz. A thick buffer oxide layer was adopted and optimized using the heat transfer physics of the finite element method to shield the TEG layers from photothermal damage during harsh laser delamination (see Fig. 3f). Figure 3g presents an SEM image showing the a-Si exfoliation layer on the bulk substrate after the first and second laser shots; sequential laser irradiation of the a-Si layer led to the generation of numerous polycrystalline Si nanoparticles.

Figure 3h shows a schematic of the GaN-based inorganic multilayer exfoliation step from the mother sapphire substrate via nitrogen gas volume expansion between the GaN layer and sapphire wafer using KrF laser irradiation [127]. A high-purity GaN LED layer on a thick Cu/Ni foil was used to fabricate flexible vertical LEDs (f-VLEDs). As shown in Fig. 3i, ultrathin, transparent, and flexible, blue-colored f-VLEDs with high optical power, a long lifetime, and excellent thermal/mechanical stability can be conformally attached to a human fingernail.

Due to the requirement of intense light intensity for lift-off processes, they have been mostly implemented by concentrating a high-energy pulsed laser beam into a small focused area, which makes it difficult to improve their scalability for mass production. Recently, lift-off technology has been demonstrated using a flash lamp light source, which possesses strong potential to enable cost-effective and large-area delamination processes for energy applications. Liu et al. used a microsecond timescale photonic lift-off (PLO) technique to implement ultrathin, flexible solar cells with a thickness of < 20 μm [103]. First, CuInSe_2_ nanocrystal-based photovoltaic devices (PVs) were fabricated on a thin polyimide (PI) layer on a metal light-absorbing layer (LAL)/rigid glass substrate. During the rapid, scalable flash lamp-based PLO process, the light pulse reached the LAL through the backside of the glass support. Subsequently, the generated heat facilitated the exfoliation of PVs on the ultra-flexible PI layer (see Fig. 3j). Figure 3k shows the calculated temperature change at the LAL-PI interface and PI surface using the SimPulse program based on the geometry of a 20 μm-thick PI on LAL-coated glass and a light pulse of 150 μs. Unlike the temperature profiles of the PI surface, the temperature at the LAL-PI interface rapidly rises and falls within a short time (approximately 550 μs), leading to the peeling-off of devices on a thin PI layer.

### Crystallization (Laser)

Laser–material interaction technologies that transform photonic energy into thermal energy have been envisioned as promising alternatives to conventional furnace heat treatment [72, 129–135]. When the laser beam is irradiated to the poor crystalline materials, the photon energy is primarily absorbed through optical absorption, which heats the material even beyond its melting point. The molten material begins to rapidly lose heat to its surroundings, causing sequential dynamics for crystallization, including nucleation, crystal growth, and formation of crystal structure: i) Small crystal nuclei form within the molten material for the transition from an unstable molten state to a stable crystalline state. ii) The molten material around the nuclei gradually attaches to them, allowing the crystals to grow. The growth rate depends on the cooling rate, thermal properties of the material, and its chemical composition. iii) As crystal growth progresses, the material forms larger crystal by transitioning its structure from the original poor crystalline to a regular crystalline lattice. The size and shape of the crystals are heavily dependent on the initial nucleation conditions and growth rate, which can be controlled by laser parameters such as optical power, pulse width, and scanning speed. Consequently, the laser-irradiation-based photothermal annealing facilitates controlled solidification and crystallization of materials such as inorganics, amorphous metals, and perovskites, which affect their material’s grain size, crystallinity, structural purity, density, and surface roughness. The modification of these key factors provides effective solutions for improving the performance and efficiency of energy applications.

**Fig. 4:**
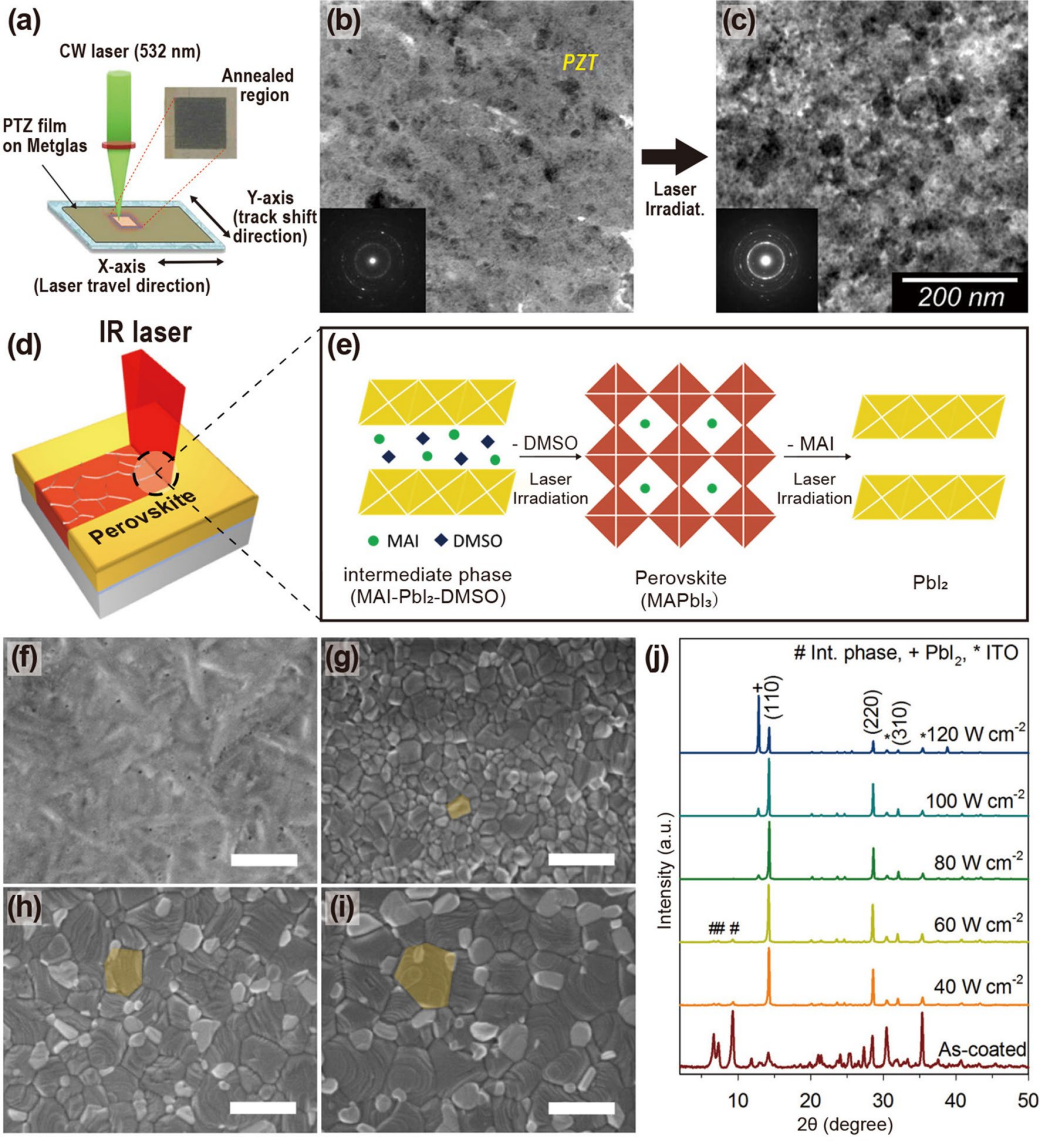
**a** Schematic diagram of CW laser irradiation process for annealing inorganic PZT film onto Metglas foil. Bright-field TEM images of **b** as-prepared PZT film and **c** laser-treated film with a laser fluence of 390 J mm^−2^; insets indicate selected area electron diffraction patterns corresponding to each TEM image. Reproduced with permission from Ref. [104]. Copyright 2016, American Ceramic Society. **d** Illustration of laser-based crystallization of MAPbI_3_ hybrid films for implementing flexible perovskite solar cells; **e** graphic illustration of phase transition steps during interaction of laser and perovskite MAPbI_3_; **f–i** SEM images of perovskite films annealed by IR laser with power densities from 0 to 100 W cm^−2^ (f: as-deposited film, g: 40 W cm^−2^, h: 80 W cm^−2^, i: 100 W cm^−2^); **j** XRD patterns obtained from annealed perovskite films by laser-induced photothermal interaction. Reproduced with permission from Ref. [97]. Copyright 2016, American Chemical Society

The schematic illustration in Fig. 4a shows the annealing process of an inorganic PZT film onto an amorphous Metglas substrate [104]. A 2-μm-thick piezoelectric PZT film deposited using granule spray in vacuum (GSV), a room-temperature deposition process, was heat-treated using a CW diode-pumped Nd:YAG thin-disk laser with a wavelength of 532 nm. Figure 4b, c shows the microstructure of the PZT films on the Metglas foil before and after CW laser annealing. The as-deposited PZT film exhibited a low crystallite fraction (Fig. 4b) and undefined diffraction rings with a few singular spots (inset). In contrast, the laser-annealed PZT film irradiated with a laser fluence of 390 J mm^−2^ exhibited improved crystallinity and grain growth (Fig. 4c). It was also found that the dielectric and ferroelectric properties of inorganic PZT films are enhanced by introducing laser-induced photothermal interaction.

Jeon et al. [97] reported controllable laser crystallization of CH_3_NH_3_PbI_3_ (MAPbI_3_) perovskite hybrid films for solar cells using a near-infrared laser. Perovskite solar cells based on spin-coated MAPbI_3_ wet film on PEDOT:PSS/indium tin oxide (ITO)/glass substrates were laterally scanned with an Nd:YAG laser beam with a wavelength of 1064 nm (see Fig. 4d). As shown in Fig. 4e, which shows the phase transition model during the laser–material interaction, laser irradiation triggered the transformation of the poorly crystalline state (intermediate phase, left panel) of the as-prepared films into a light-absorbing crystalline structure (perovskite MAPbI_3_, center panel). A high-power density of over 120 W cm^−2^ initiated the decomposition of MAPbI_3_ into PbI_2_ (right panel) [136, 137]. Figure 4f–i shows the morphologies of the perovskite films irradiated with laser power densities ranging from 0 to 100 W cm^−2^ at a scan rate of 0.1 mm s^−1^. With increasing applied power density, the low-crystalline phase of the as-deposited film transitioned into polycrystalline structures, and the grain size increased. The drastic change in the X-ray diffraction (XRD) patterns (Fig. 4j) of the perovskite films fabricated at various laser powers indicates that the laser-induced photothermal interaction activates the transformation and thermal decomposition of tetragonal MAPbI_3_.

Achieving uniformity and high quality in crystallization is essential for optimizing the performance of energy materials and devices. However, elevating completeness of the crystallization technology to industrial-scale applications is quite challenging. For example, the low-temperature polycrystalline silicon process took two decades to be adapted for industrial use due to its variability in material properties and complex dynamics of heat and mass transfer during the process. To address these challenges, the spatial precision and controllability of LMI processes should be ensured, which are feasible by advancing optical technologies that can more accurately control the distribution of energy and time during crystallization. In addition, enhancing uniformity in material synthesis and deposition is required to improve the production yield of energy systems without deteriorating their quality and performance.

### Crystallization (Flash)

Flash lamps have garnered substantial attention owing to their rapid processing capability, highly efficient light output, and large-scale processability [70, 105, 138–141]. Xenon flash lamp-induced photonic annealing techniques enable rapid heating/cooling of energy materials to the crystallization temperature, facilitating crystallization within milliseconds without distinct radiative damage [41, 92, 114, 138, 142, 143].

**Fig. 5:**
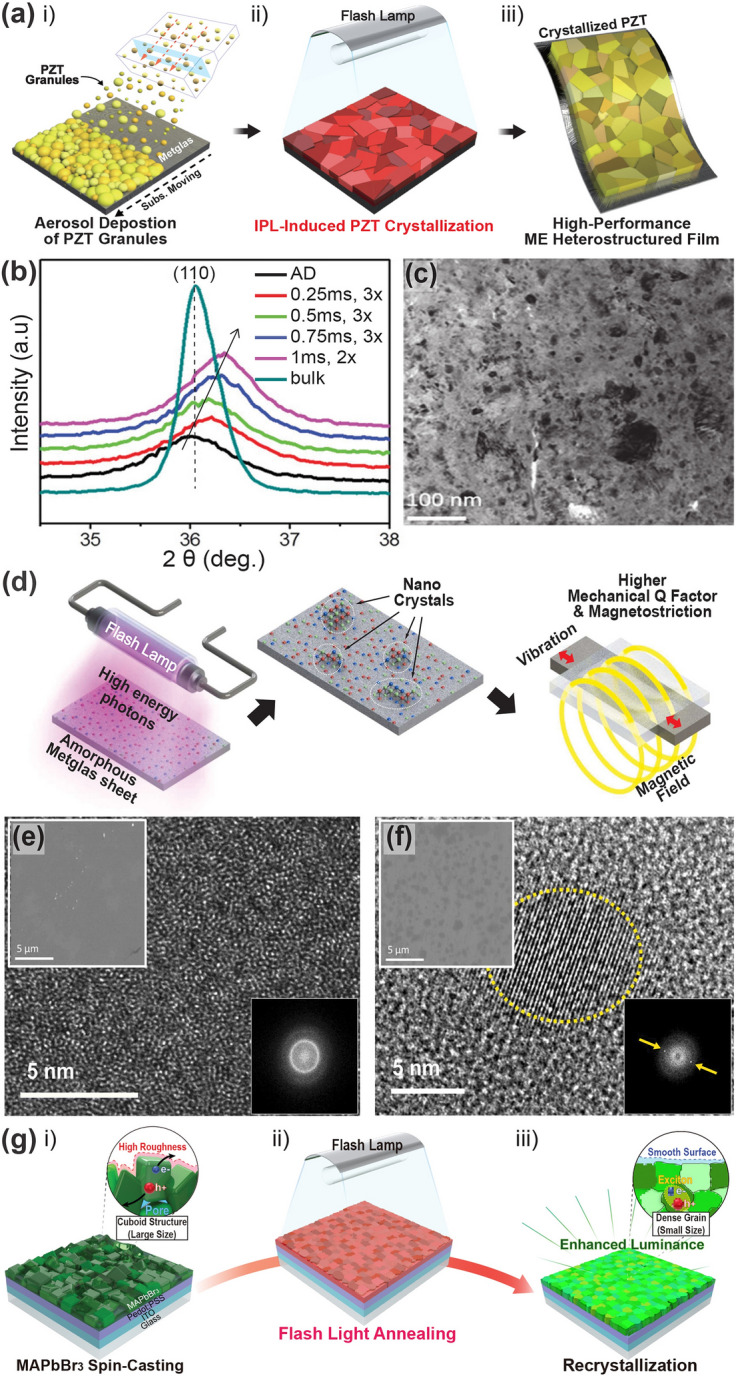
**a** Schematic illustrations describing (i) aerosol deposition process and (ii, iii) crystallization treatment using IPL thermal annealing based on xenon flash lamp for facilitating enhanced PZT-film piezo-/ferro-electric properties; **b** magnified XRD patterns around (110) peak of as-prepared and flash lamp-irradiated films with different pulse durations; **c** bright-field high-resolution transmission electron microscopy (HR-TEM) image of PZT/Metglas ME composite film. Reproduced with permission from Ref. [115]. Copyright 2023, Wiley–VCH. **d** Overall flash photon annealing procedure of amorphous Metglas sheet for nanocrystal recrystallization and improved magnetic performance. Microstructural features of **e** bare Metglas and **f** flash-annealed sheet with partial surface nanocrystallization; top, bottom insets: SEM images and Fast Fourier Transform patterns of each sample, respectively. Reproduced with permission from Ref. [144]. Copyright 2021, Elsevier. **g** Overall concept of flash lamp-based ultrafast recrystallization process of MAPbBr_3_ perovskite films; densely recrystallized perovskite film obtained by flash lamp annealing allows for a smoother surface with small grain size and improved optical properties of perovskite-based LEDs. Reproduced with permission from Ref. [145]. Copyright 2019, Elsevier

Palneedi et al. [115] fabricated a high-performance magnetoelectric (ME) composite film comprising piezoelectric and magnetostrictive films by adopting an intense pulsed-light (IPL) thermal treatment with a xenon flash lamp system. For ME heterostructured composite structure, the aerosol-deposited piezoelectric PZT thick films on a Metglas foil were attached to a glass slide and then irradiated with an IPL of various pulse durations (0.25–1 ms, as shown in Fig. 5a). The XRD patterns of the as-deposited and IPL-annealed PZT films in Fig. 5b show significant discrepancies in intensity, broadness, and peak position, indicating that the flashlight-based photothermal energy induces crystallinity enhancement, nanocrystalline formation, and residual compressive stress inside the films. The microstructure of the PZT/Metglas ME film (Fig. 5c) shows an improved crystalline structure with an increased fraction and crystallite size, compared to the as-prepared films having a mixed amorphous and crystalline microstructure.

Peddigari et al. [144] reported an ultrasensitive magnetic field detector comprising a flash photon-annealed amorphous Metglas substrate and piezoelectric composite. They used high-temperature flashlight annealing with a short pulse of ~ 300 μs to promote surface recrystallization of amorphous Metglas sheets at the nanometer scale for enhanced magnetic performance (Fig. 5d). Compared with the microstructure of the pristine Metglas film (Fig. 5e), partial recrystallization at the surface was formed on light-induced films (Fig. 5f) by employing flash photonic annealing. This novel strategy improved the magnetic flux concentration and piezo-magnetic coefficient, facilitating the development of an ultrasensitive magnetic field sensor.

Figure 5g shows the overall procedure for the flash-induced ultrafast recrystallization of perovskite crystalline structures to enhance their optoelectronic properties [145]. The perovskite MAPbBr_3_ precursor-based structures were quickly heated and quenched within milliseconds with a flash light, resulting in a densely recrystallized MAPbBr_3_ perovskite film with a fine grain size of ~ 38 nm without radiative damage. During the recrystallization process, the cuboid-structured perovskite crystals that initially formed a rough surface were heated and melted into a single dense film with smooth surface. As a result, the root mean square, indicating the surface roughness of the bare perovskite layer, decreased from 8.47 to 3.22 nm using the flash light process, thereby achieving a higher current efficiency of perovskite-based LEDs compared to typical thermal annealing processes.

### Sintering (Laser)

Laser sintering techniques are gaining attention for manufacturing nanostructures (in hierarchical structures and patterning, for example) in energy device applications such as batteries, solar cells, supercapacitors, and electrocatalytic electrodes. These processes allow us to engineer materials with highly specific structures, such as tailored porosity and complex geometries, which are essential for enhancing the performance of energy systems.

**Fig. 6:**
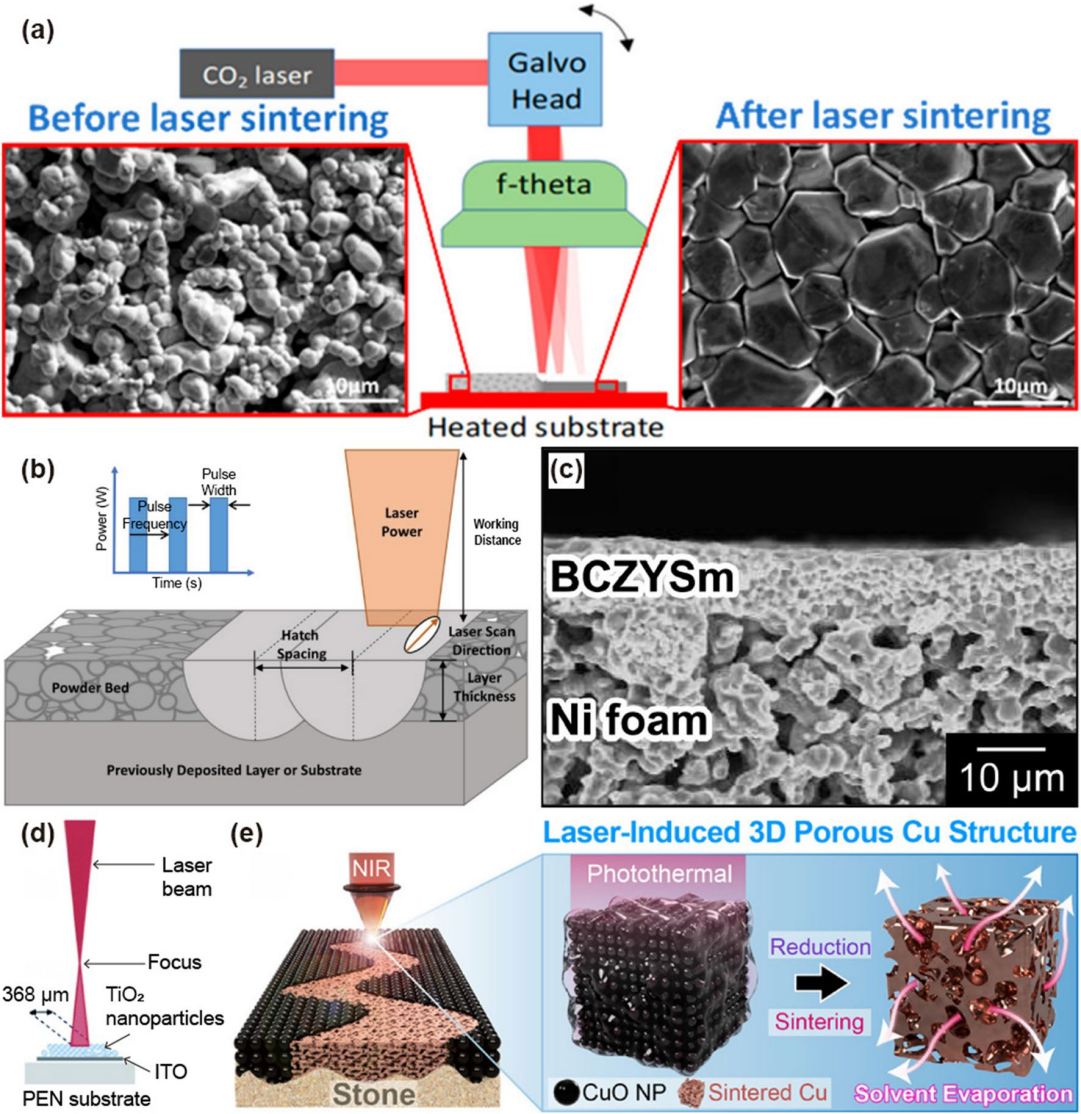
Schematic of **a** CO_2_ laser sintering of LLZTO films. Reproduced with permission from Ref. [160]. Copyright 2022, ACS. **b** Selective laser sintering and laser pulse profile. Reproduced with permission from Ref. [161]. Copyright 2021, Elsevier. **c** Cross-sectional SEM image of BCZYSm on nickel foam. Reproduced with permission from Ref. [162]. Copyright 2021, Elsevier. **d** Schematic of laser beam irradiation on TiO_2_ film grown on ITO-coated PEN substrate. Reproduced with permission from Ref. [163]. Copyright 2014, RSC. **e** Laser-induced explosive reduction and sintering of CuO nanoparticles for 3D, porous Cu electrode structure on natural stone surface. Reproduced with permission from Ref. [98]. Copyright 2022, ACS

Among the solid-state electrolytes (SSEs), garnet-type materials [Li_7_La_3_Zr_2_O_12_ (LLZO) and Li_6.4_La_3_Zr_1.4_Ta_0.6_O_12_ (LLZTO)] have attracted attention owing to their superior chemical compatibility with Li metal, high ionic conductivity, and layered oxide cathodes [146–153]. However, the LLZTO garnet-type electrolytes possess drawbacks, such as poor sinterability, high sintering temperatures (above 1100 °C), long soaking time of 10–40 h, and high packing density, which makes these SSEs less suitable for battery applications [146, 152, 154–158]. In addition, Li loss can occur owing to its volatilization, forming a secondary phase (La_2_Zr_2_O_7_) from the decomposition of LLZTO, resulting in a porous microstructure and degradation of mechanical and electromechanical responses [155, 159]. Erika et al. [160] utilized a CO_2_ laser sintering process for the densification of garnet-type SSE LLZTO films, as schematically shown in Fig. 6a. A CO_2_ laser was used to sinter successive layers of powder by heating it to just below its boiling point, which fuses the particles in the powder into a solid form. This approach has reduced Li loss by rapid sintering (< 1 s), which facilitates unique anisotropic shrinkage that decreases film thickness, and creates a wave-like surface topology that allows three-dimensional (3D) interfacial contact with the electrode materials. In addition, the laser-sintered LLZTO films exhibited highly dense, crack-free, homogeneous microstructure with outstanding electrical properties (high conductivity of 0.26 mS cm^−1^ and low activation energy of 0.08 eV).

Selective laser sintering (SLS) is a powder-based 3D printing technology that uses a laser as the power and heat source to fuse tiny particles of polymer/ceramic powder material to form a solid structure. SLS has been developed fabricating electrodes in Li-ion batteries (LIBs), enabling LIBs to have higher performance than that achieved with conventional electrode fabrication methods such as thin film deposition and roll-to-roll (R2R) processing [164]. Katherine et al. [161] utilized SLS to fabricate binder-free lithium nickel cobalt aluminum oxide (NCA) cathodes for LIBs (Fig. 6b). NCA is primarily utilized as a cathode material that enables high energy and power densities for use in electric vehicles. Layer-by-layer SLS of bulk 3D NCA samples were refined using a parametric single-track analysis, resulting in a dual phase of layer (*R 3 m* symmetry) and rock salt structure (*Fm-3 m*) with porous structure with a grain size of 2–3 µm. Retaining the electrochemically active layered structure and porous morphology in the NCA samples enabled the construction of binder-free, 3D-printed cathodes for next-generation LIBs with enhanced power densities.

BaZrO_3_–BaCeO_3_-based electrolytes were fabricated by a rapid laser sintering process for application in ceramic fuel cells and electrolyzers [165, 166]. However, during wet processing/sintering, chemical reactions between ceramic elements (BaZrO_3_) and polar solvents [Ba(OH)_2_, BaCO_3_] lead to volume changes that crack the electrolytes [167]. To address such problems, Akihiro et al. [162] utilized chemically inert saturated-hydrocarbon with proton-conducting ceramic (BCZYSm: BaCe_0.7_Zr_0.1_Y_0.07_Sm_0.13_O_3-δ_)-based slurries composed of hexadecane, polybutene, and fatty acids and employed rapid laser sintering at 600 °C over a short period (~ 3 s). Figure 6c shows the cross-sectional SEM image of a laser-sintered BCZYSm membrane. It revealed a dense and uniform microstructure with a thickness of 13.5 µm and proton conductivity of 10^–4^ S/cm. This study offered a framework for developing proton-conducting ceramic membranes via rapid laser sintering and the use of hydrophobic slurries.

Flexible solar cells have received considerable attention in the photovoltaic market owing to their light weight, low cost, ease of fixation on complex surfaces, and good compatibility [168]. Flexible substrates, including metal foils or meshes, conductive plastic films, and ITO-coated polyethylene naphthalate (PEN)/PET, are utilized for flexible dye-sensitized solar cell (DSC) applications [169]. Although nontransparent metal substrates require backside lighting, the counter electrode can absorb unwanted light, and the electrolyte is an issue for this type of solar cell. The efficiency of plastic substrate-based DSCs is 7%-8%, which is lower than that of conventional DSCs on fluorine-doped tin oxide (FTO) glasses [170, 171]. This is attributed to the high sintering temperature (~ 550 °C) of the TiO_2_ film grown on the FTO glass. The ITO-coated PEN and PET substrates can withstand heat below 150 °C. Liqun et al. [163] fabricated TiO_2_ films on conductive plastic substrates using the SLS method to improve the structural and physical properties, as well as the efficient charge collection, of solar cells operating at low temperatures, thereby achieving high-performance, flexible DSCs. A schematic of the laser-beam irradiation of the TiO_2_ nanoparticle film is shown in Fig. 6d. The absorbed laser energy supports electrical contact between the TiO_2_ nanoparticles without damaging the plastic substrate. In addition, the SLS technology reduces the electron transport resistance and enhances the recombination resistance of the TiO_2_ film, leading to an improvement in the charge collection efficiency of the DSC.

LMI-induced sintering technologies have been extensively used to fabricate electrical components for energy devices [172, 173]. Back et al. [98] developed the SLS technique to construct the electrodes of microsupercapacitors (MSCs) on a natural stone surface, as shown in Fig. 6e. Interdigitated Cu conductors with hierarchical nano-/microvoids were successfully fabricated on a rough stone surface by applying a laser-induced explosive reduction and sintering (ERS) process to CuO nanoparticles. Highly capacitive and faradaic metal oxides (Mn_3_O_4_ and Fe_3_O_4_) were sequentially grown on porous Cu electrodes using an electroplating method to fabricate the cathode and anode materials for the hybrid MSC. The Mn_3_O_4_ and Fe_3_O_4_ layers were changed into sponge-like and hierarchical coral structures at a load mass of 75 μg, which improved the surface area and output performance of MSC. The hybrid MSC displayed a high energy and power density of 6.55 μWh cm^−2^ and 1.2 mW cm^−2^, respectively.

### Sintering (Flash)

Flash light with a wide spectrum of photon energies can be optically absorbed by various types of materials, including ceramic, metallic, and carbon nanomaterials for sintering and annealing, rapidly increasing the temperature in milliseconds. The broad range of wavelengths ensures that different components of composite materials absorb the relevant flash light wavelengths, leading to uniform processing and improved material properties. For example, in the sintering of metallic NWs, the wide spectrum of flash lamps facilitates comprehensive heating and effective fusion of the NWs, resulting in high-conductivity transparent films [39]. The flash-induced precise control over microstructural features enables optimal electron and ion transport pathways, significantly improving performance and reliability of energy conversion and storage applications [174–179].

**Fig. 7:**
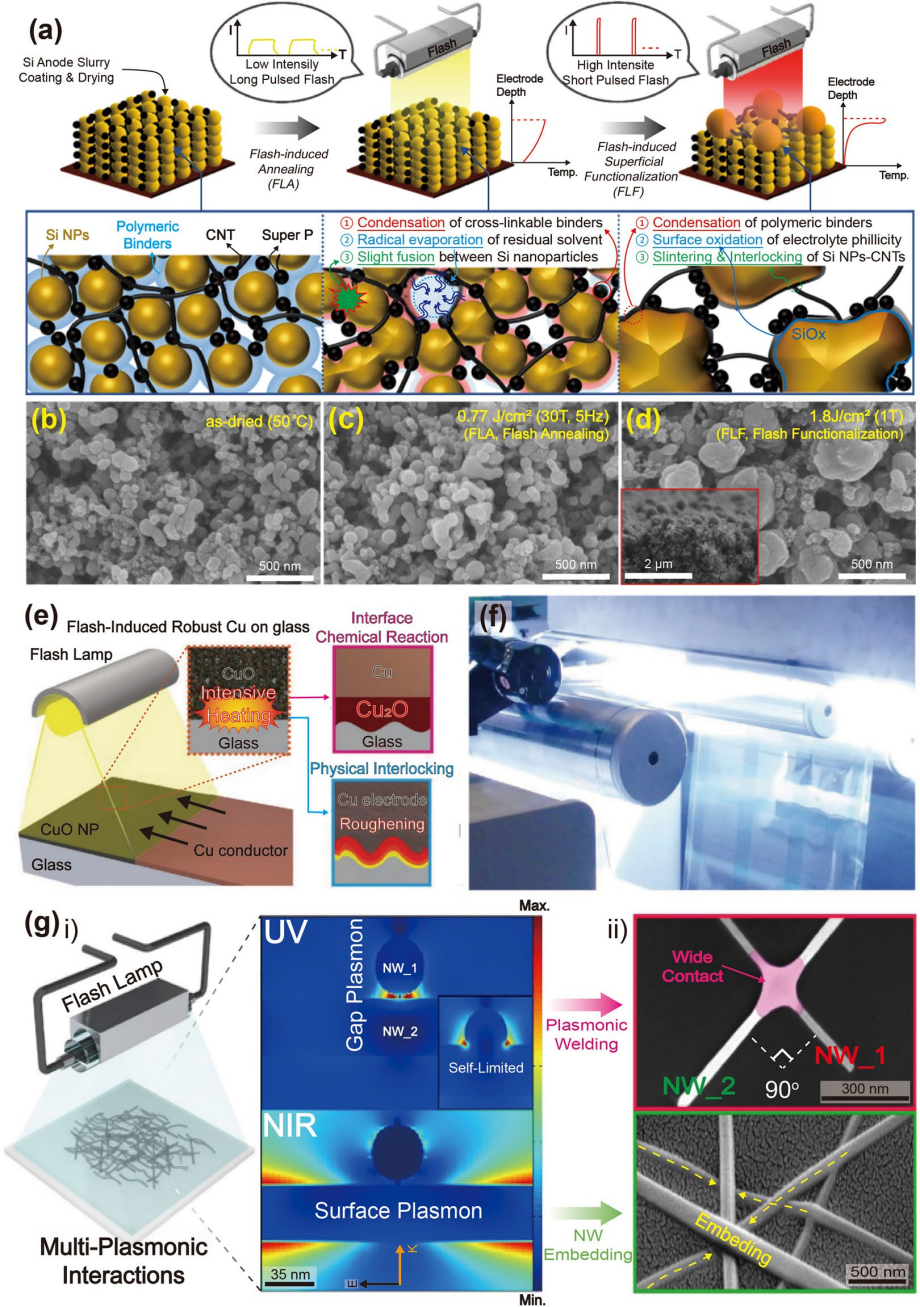
**a** Schematic of fabrication steps for Si anode flash irradiation and SEM images of Si anode captured under **b** as-dried, **c** flash-annealed, and **d** flash-functionalized conditions (**Fig. 7d** inset shows cross-sectional SEM image). Reproduced with permission from Ref. [93]. Copyright 2021, ACS. **e** Schematic of Cu electrode flash irradiation on glass substrate for μLED interconnection. Reproduced with permission from Ref. [76]. Copyright 2021, Wiley–VCH. **f** Photographic image of photonic sintered silver grid structures during flash. Reproduced with permission from Ref. [92]. Copyright 2012, Wiley–VCH. **g-(i)** Schematic of AgNW flash irradiation for transparent, flexible energy harvester and 3D finite-difference time-domain analysis; **g-(ii)** Plane view (top) and SEM image surface morphology (bottom) of welded AgNWs on PET film. Reproduced with permission from Ref. [114]. Copyright 2016, Wiley–VCH

The commercialization of Si-based anode materials for LIBs has been limited because of their poor cycling stability, which is caused by a significant volume change (~ 300%) during the charge–discharge process. Seok et al. [93] demonstrated a flash-induced Si anode to improve the cycling stability of high-performance LIBs. The overall flash annealing process for the Si anode of a LIB is shown schematically in Fig. 7a. The Si anode materials were synthesized using a conventional fabrication method. The slurry, consisting of Si nanoparticles, conductive additives (carbon black and carbon nanotubes in a weight ratio of 3:1), and cross-linkable binders (carboxymethyl cellulose and polyacrylic acid mixed in a 1:1 weight ratio), was used to coat the Cu foil and dried at 50 °C. The prepared Si anodes were irradiated by a flash light under two irradiation conditions: low-intensity and long-pulsed (LILP) and high-intensity and short-pulsed (HISP). The Si anode irradiated by the LILP flash induces a slight, uniform increase in temperature around the anode region, known as the flash light-induced annealing (FLA) process, resulting in binder condensation, residual solvent evaporation, and slight fusion. This improved the cycling stability by increasing the binding strength between the anode materials and minimizing crack formation caused by Si anode volume variations. When the Si anode was irradiated by the HISP flash, the high temperature generated around the anode led to flash light-induced functionalization (FLF). The FLF process caused large pore formation by sintering Si nanoparticles and carbon nanotubes, which improved the Si anode rate capability by facilitating Li ion and electron transport. Figure 7b–d shows the Si anode SEM images under as-dried, FLA, and FLF conditions (the cross-sectional SEM image is presented in the inset). Slight fusion between Si nanoparticles was observed in the FLA-treated Si anode (Fig. 7c). In contrast, the Si nanoparticles were completely sintered, resulting in larger particles with a diameter of 300–500 nm after the HISP flash light (Fig. 7d). It was concluded that the Si anode sintered particle size and surface structure could be controlled by varying the irradiation energy density and pulse width. The study suggests a low-cost method for improving battery electrode performance via ultrafast flash irradiation, replacing the traditional long-term thermal treatment.

Cu electrode and glass substrate materials are attractive for thin-film micro-LEDs (μLEDs) for display applications [180–183]. The concerns with thin-film μLEDs are mostly related to the substrate and electrodes [184, 185]. Weak Cu adhesion on glass substrates causes electrode delamination under mild temperature and humidity fluctuations, breaking current-driven μLEDs [186]. Jung et al. [76] proposed a robust Cu electrode on a glass substrate for fabricating AlGaInP thin-film LEDs using a flash lamp. The flash-induced Cu electrode on a glass substrate for LEDs is illustrated in Fig. 7e. The CuO nanoparticles were spin-coated onto a glass substrate and subsequently transformed into a Cu film by flash light irradiation, producing reductive sintering reactions. Concurrently, an ultrathin Cu_2_O interlayer was created between the glass and Cu electrodes for robust adhesion. In addition, physical nano-interlocking of the Cu electrode occurs via glass softening and nanoscale roughening. The flash-induced Cu electrode has an adhesion energy of 10 J m^−2^, which is five times higher than that of a conventionally grown Cu electrode.

Dechan et al. [92] fabricated Ag grids on a flexible PET film using photonic sintering for an ITO-free polymer solar cell module. A diagonal Ag grid pattern of 600 × 600 dots per inch was printed on a flexible PET substrate using aqueous silver nanoparticle ink and an R2R inkjet printer. Figure 7f shows the R2R photonic sintering of the inkjet-printed silver nanostructures. The xenon flash lamp was discharged at a pulse energy of 830 J at 0.5 ms duration. This photonic sintering considerably enhanced both adhesion and output performance (low sheet resistance of 9–12 Ω sq^−1^) of the Ag grid.

A flashlight sintering method was adopted for 1-D Ag NWs to develop transparent and flexible conducting materials for energy applications. Park et al. [114] utilized a wide range of flash spectra to enable instantaneous multi-plasmonic interactions of Ag NWs for transparent flexible electrodes, as shown in Fig. 7g. The intense xenon flash light spectrum near 400 nm wavelength-induced localized and self-limited plasmonic heat generation (photothermal reactions) at NW junctions, which led to fully welded Ag NWs with outstanding performance (low resistance of 5 Ω sq^−1^, high transparency of 90%, and strong adhesion of 30.7 J m^−2^). The NIR spectra of the flash light locally melted the interface between Ag NWs and PET by inducing surface plasmon polarization, thus reinforcing the adherence of the Ag NW layer by 310% compared to that of the pristine Ag NW/PET film.

### Surface Texturing and Modification (Laser + Flash)

Laser- and flash-induced surface modifications of materials have been reported for energy conversion/storage applications such as solar cells, fuel cells, LIBs, and triboelectric nanogenerators. By utilizing the strong, instantaneous light energy emitted from lasers or flash lamps, the precise and physical removal or texturing of selective surface areas can be achieved without altering chemical structure of target materials. This photothermal effect can be used to enhance the properties of energy materials, and surface area of energy applications, leading to improved device performance and efficiency.

**Fig. 8:**
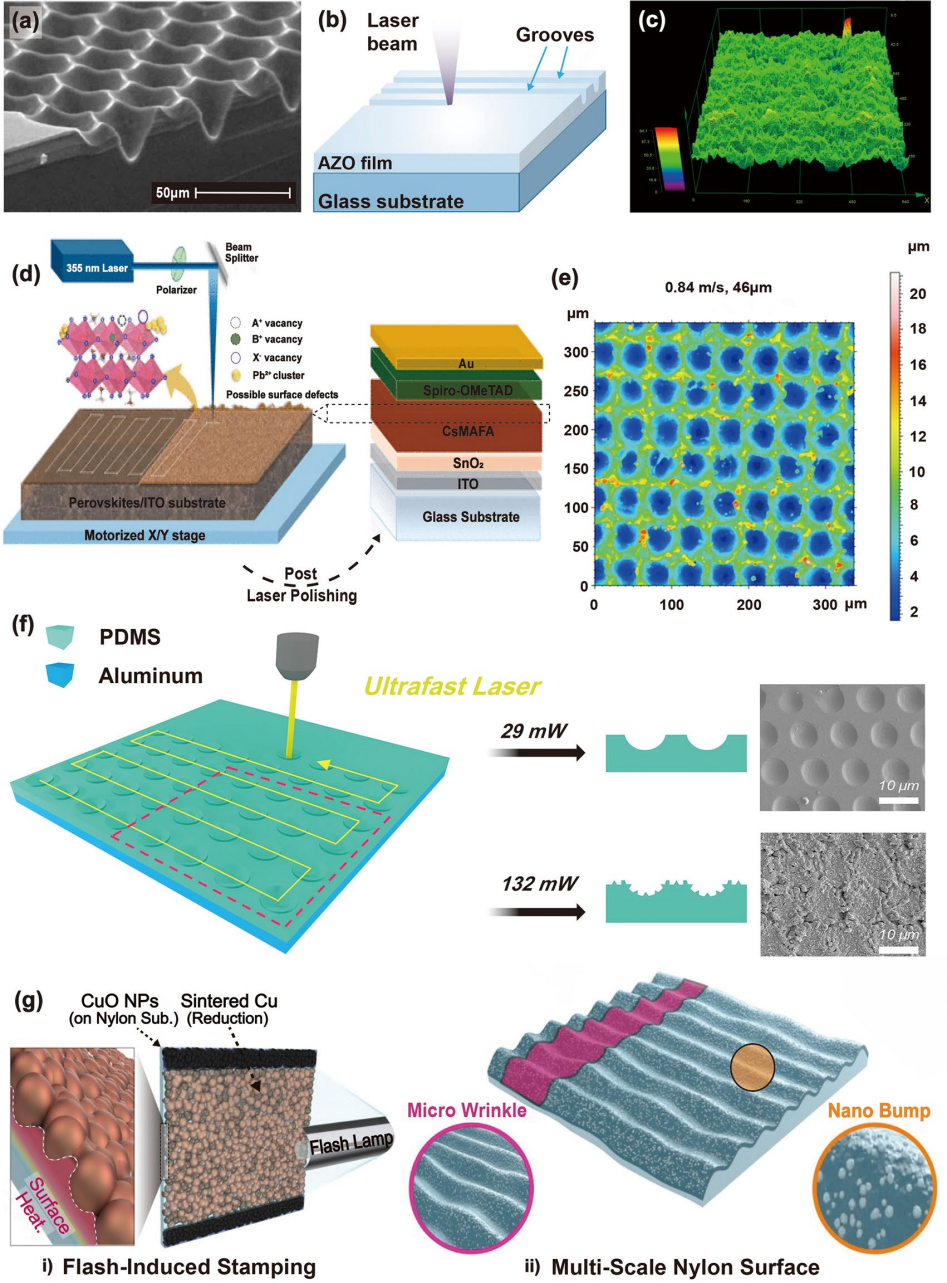
**a** Cross-sectional SEM image of laser-ablated texture. Reproduced with permission from Ref. [187]. Copyright 2006, Wiley–VCH. **b** Schematic of laser-texturing ZnO:Al thin films. Reproduced with permission from Ref. [188]. Copyright 2019, Elsevier. **c** Surface roughness morphology of modified anode substrate. Reproduced with permission from Ref. [189]. Copyright 2018, Elsevier. **d** Schematic of laser setup indicating raster scanning of metal halide perovskite thin film surface (left side) and laser-polished thin film utilized for solar cell fabrication (right side). Reproduced with permission from Ref. [190]. Copyright 2023, ACS. **e** Surface topography of laser-textured Al CCs surface denoted by a green circle. Reproduced with permission from Ref. [58]. Copyright 2023, Elsevier. **f** Schematic of PDMS surface-patterning method using ultrafast laser irradiation (left side) and SEM images of laser-irradiated PDMS at 29 and 132 mW laser power (right side). Reproduced with permission from Ref. [191]. Copyright 2017, Elsevier. **g** Schematic of flash-induced fabrication process for MMTENG. Reproduced with permission from Ref. [94]. Copyright 2020, Elsevier

Texturing of solar cells aims to reduce reflection on the front surface and enhance light trapping. However, the difficulty in appropriately texturing the front surface is a key performance restriction using multi-crystalline silicon. Abbott et al. [187] prepared laser-texture-processed solar cells with higher efficiency. Isotropic laser ablation process was conducted to shape the silicon wafer surface, independent of the crystallographic orientation. Laser ablation (Q-switched and Nd:YAG lasers) sculpts the surface of the silicon, removing the bulk of the silicon necessary for texturing. Figure 8a shows a tilted-view SEM image of the laser-ablated pits organized in the interlocking pattern. Chemical etching was performed to remove the slag, smoothen the surface, and eliminated laser damage. Thus, laser texturing appears a promising for texturing multicrystalline and monocrystalline silicon solar cells and enhancing their efficiency.

Similarly, Canteli et al. [188] reported that laser texturing of ZnO enhanced the light scattering and output current response of Al thin film-based solar cells by producing simple patterns via direct laser scribing on a transparent conductive oxide surface (Fig. 8b). The laser texturing procedure not only provides an eco-friendly solution compared to plasma or chemical etching processes but also enables selective patterning of wide-ranging materials regardless of their crystallographic structure.

Figure 8c shows the surface roughness morphology of the NiO-yttrium stabilized zirconia anode substrate, which was modified using the laser-machining technique. Laser texturing increased the contact area of the electrode–electrolyte interface, enhancing the solid oxide fuel cell's power density by 47% [189]. The improved power density is directly correlated with fuel cell efficiency and longevity, highlighting the potential of laser texturing as a simple, sustainable technique for optimizing energy storage device performance.

Interface engineering is a common approach for reducing surface defects in perovskite solar cells to enhance their open-circuit voltage and efficiency. Kedia et al. [190] proposed polishing a metal halide perovskite thin-film surface using a nanosecond-pulsed ultraviolet laser to diminish surface defects (e.g., dangling bonds, secondary phases, and suboptimal stoichiometry). Figure 8d shows a schematic of the laser process used to modify the surface of the perovskite thin film for solar cell applications. At a laser wavelength of 355 nm, the perovskite thin films have a high absorption coefficient, resulting in a penetration depth of a few tens of nanometers. The laser polishing technique successfully reduced surface defects of the perovskite film, such as dangling bonds, suboptimal stoichiometry, and undesirable phases, which can create nonradiative recombination centers and subsequently decrease the efficiency of photogenerated carriers. By minimizing these defects through the control of the laser fluence and scanning speed, the photophysical properties of the perovskite thin film surface were effectively enhanced without changing its thickness. The resulting solar cell achieved an enhanced performance by improving the hole transport interface and modifying the surface recombination losses.

Ravesio et al. [58] applied nanosecond-pulsed laser texturing of Al current collectors (CCs) to enhance the electrochemical performance of LIB-based cathodes. The surface topography of the laser-textured Al CCs is shown in Fig. 8e. The optimized laser processing conditions (laser pulse fluence of 25 J cm^−2^, scanning speed of 0.84 m s^−1^, and hatch distance of 46 µm) improved surface area and LIB electrode performance. This laser treatment also improved the adhesion between the active materials and current collectors.

Triboelectric nanogenerators (TENGs) have been explored to convert ambient mechanical energy into electricity for energy harvesting applications [192, 193]. A femtosecond laser was employed to create direct patterning on the polydimethylsiloxane (PDMS) surface to improve the output power [191]. The PDMS surface-patterning method using ultrafast laser irradiation (left side) and SEM images of the laser-irradiated PDMS at laser powers of 29 and 132 mW (right side) are shown in Fig. 8f. The SEM image of the PDMS laser-irradiated at 29 mW revealed a regular and well-ordered concave hemispherical surface morphology. In contrast, a laser power of 132 mW induced a deep and irregular sub-morphology. The regular and well-ordered microstructures on the PDMS surface by laser irradiation enhanced the contact area between triboelectric and metal layers, resulting in a high-power density of 107.3 μW cm^−2^, which is almost two times higher than that resulting from conventional surface modification.

A flash-induced multiscale magneto-mechano-triboelectric nanogenerator (MMTENG) was developed to fabricate self-powered flexible LEDs for optogenetic neuromodulation applications [94]. In Fig. 8g, the flash-induced multiscale MMTENG surface-texturing process is schematically illustrated. Light-absorbing CuO nanoparticles were coated onto the triboelectric nylon film to act as a heating amplifier under flash light exposure. The millisecond flash light irradiation of the CuO/polymer surface induced a nanoscale bumpy texture and a microscale wrinkle structure via multiscale interface interactions, enhancing the active friction area and output performance of the MMTENG.

### Carbonization (Laser + Flash)

Laser- and flash-induced carbonization effects have been widely explored for electronic and energy-storage device applications by successfully demonstrating a variety of carbon-based materials, including carbon fibers, carbon nanotubes, graphene, and graphite. Carbonization is the conversion of organic materials into carbon or carbon-containing residues by heating them in a controlled environment, typically characterized by high temperatures and restricted oxygen availability. These conditions for carbonization can be efficiently achieved through the instantaneous LMI characteristic.

**Fig. 9:**
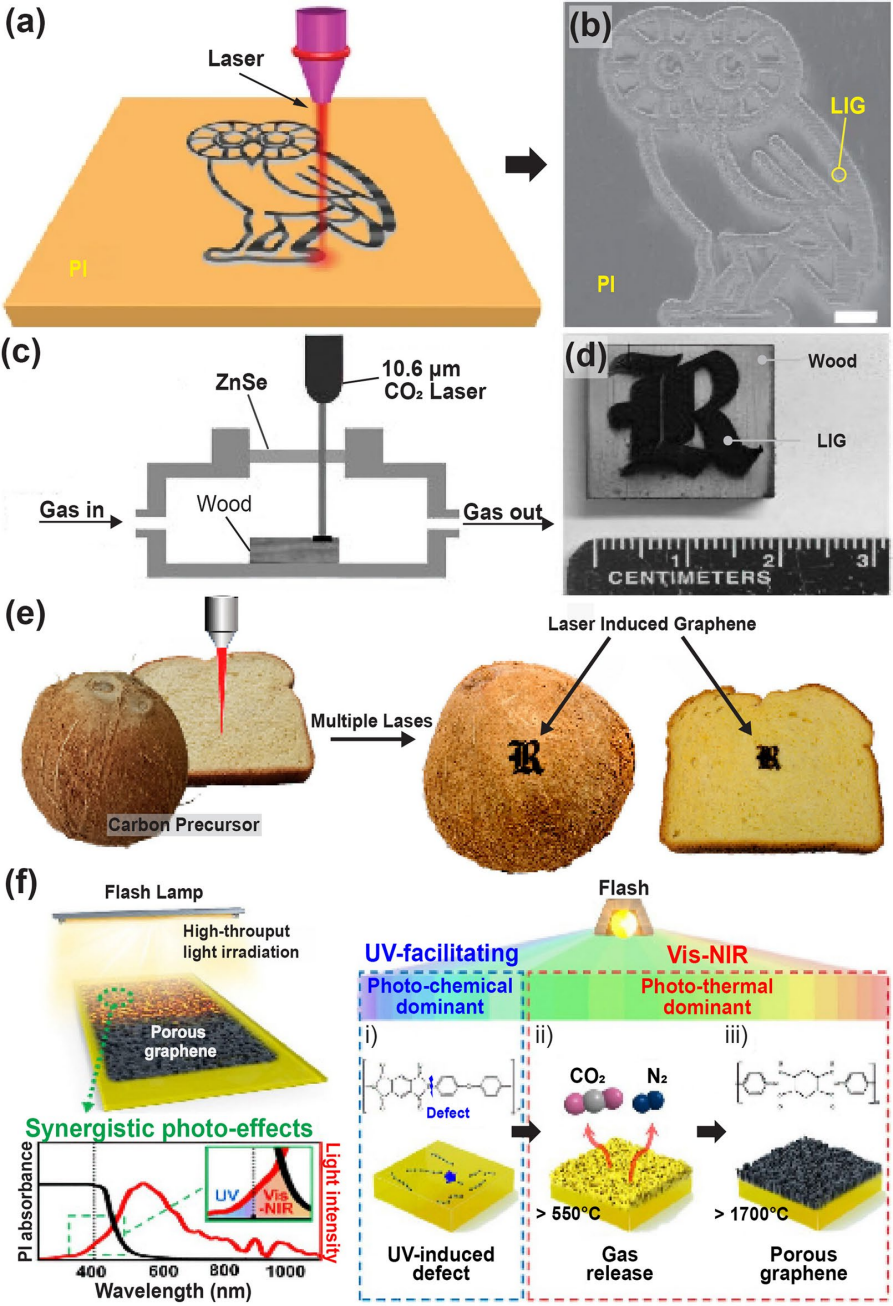
**a** Schematic illustration of laser-induced graphene synthesis from polyimide film. **b** SEM image of laser-induced graphene pattern. Reproduced with permission from Ref. [99]. Copyright 2014, Springer Nature. **c** Schematic of pine wood converted into hierarchical porous graphene using CO_2_ laser scribing. **d** Photographic image of laser-induced graphene patterned into a logo on wood. Reproduced with permission from Ref. [194]. Copyright 2017, Wiley–VCH. **e** Laser-induced graphene on substrates such as coconut and bread. Reproduced with permission from Ref. [195]. Copyright 2018, ACS. **f** Schematic of the overall flash-induced graphene fabrication process. Reproduced with permission from Ref. [197]. Copyright 2023, Springer Nature

Jian et al. [99] developed a scalable approach to patterning of porous graphene films from commercial polymers using a CO_2_ laser. A commercial polyimide film was irradiated with a CO_2_ infrared laser under ambient conditions to produce a porous graphene film called laser-induced graphene (LIG, Fig. 9a). As shown in the SEM image in Fig. 9b, LIG can be easily written into diverse designs (owl shape) using computer-controlled laser scribing. This approach is scalable and economical for fabricating large-area devices and can be applied to roll-to-roll manufacturing processes.

Ruquan et al. [194] used CO_2_ laser scribing to transform wood into hierarchical porous graphene (Fig. 9c). The pine wood was carbonized into 3D porous graphene by irradiating a CO_2_ laser (wavelength 10.6 µm) under an Ar/H_2_ atmosphere. LIG patterns on a wood surface can be readily and quickly fabricated into numerous shapes/devices using a computer-controlled design. As shown in Fig. 9d, the logo of the letter R was patterned using laser scribing. LIG with an expansive surface enables enhanced electrochemical interactions at the material interface, offering considerable advantages for developing high-efficiency energy-storage devices.

Moreover, multiple-pulse laser scribing has been utilized to convert various types of substrates (renewable precursors: food, cloth, paper, and cardboard) into LIG [195]. As shown in Fig. 9e, the coconut surface and bread were modified to LIG with the letter R by irradiation using a CO_2_ laser. Unlike the polymer precursors reported to produce LIG, these renewable precursors are plentiful, cost-effective, and biodegradable. The ability to design LIG on these renewable precursors is promising for application in flexible MSCs. Zhu et al. [196] developed a simple method to fabricate flexible graphene–copper composites (GCCs) by combining laser irradiation and efficient electrodeposition. The PI film is exposed to an ultrafast picosecond laser in order to precisely and selectively create the required LIG region with sufficient electrical conductivity. The electrodeposition stage creates the localized deposition of copper atoms, resulting in the formation of the flexible GCC with a spatial pattern defined by the laser.

Lee et al. [197] reported flash-induced porous graphene (FPG) with excellent electromagnetic interference shielding performances for drones and wearable energy-shielding applications. An overall concept of the FPG fabrication process is shown in Fig. 9f. The UV region of the broad-spectrum flash light breaks the chemical bonds in the polyimide molecules (photochemical reaction), resulting in defects that allow for efficient light absorption in the visible–near-infrared (Vis–NIR) wavelengths. The photothermal energy generated by Vis–NIR-range flash light led to graphene formation, resulting in the production of a large-area (5 × 10 cm^2^) graphene with a low density of 0.0354 g cm^−3^ in a few milliseconds. This FPG exhibited outstanding electromagnetic interference shielding effectiveness of 1.12 × 10^5^ dB·cm^2^ g^−1^ because of its low sheet resistance of 18 Ω sq^−1^ and porous structure with high internal scattering capability.

### Chemical Reaction (Laser)

Laser irradiation process enables heating of energy materials up to extremely high temperatures within milliseconds, showing a substantial potential to provide efficient, effective, and precise strategies for photo-thermochemical reactions such as oxidation, reduction, and doping. Oxidation and reduction are specific types of chemical reactions that focus on the transfer of electrons between substances. Oxidation involves the loss of electrons or an increase in the oxidation state, typically resulting in an increase in positive charge or a more oxidized state. Doping process involves the addition of very small amounts of foreign elements (impurity atoms) into an existing bulk substance, allowing these atoms to diffuse into the material’s matrix. Unlike synthesis process, this method can modify the material’s electrical, optical, and electrochemical properties without significantly changing the basic chemical structural frameworks of the original materials. For instance, heteroatoms like boron, nitrogen, and sulfur can be diffused into electrode materials through photon-induced doping process, altering various kinds of electrochemical properties of energy devices, such as electrode's affinity, and charge storage capacity/stability/efficiency.

**Fig. 10:**
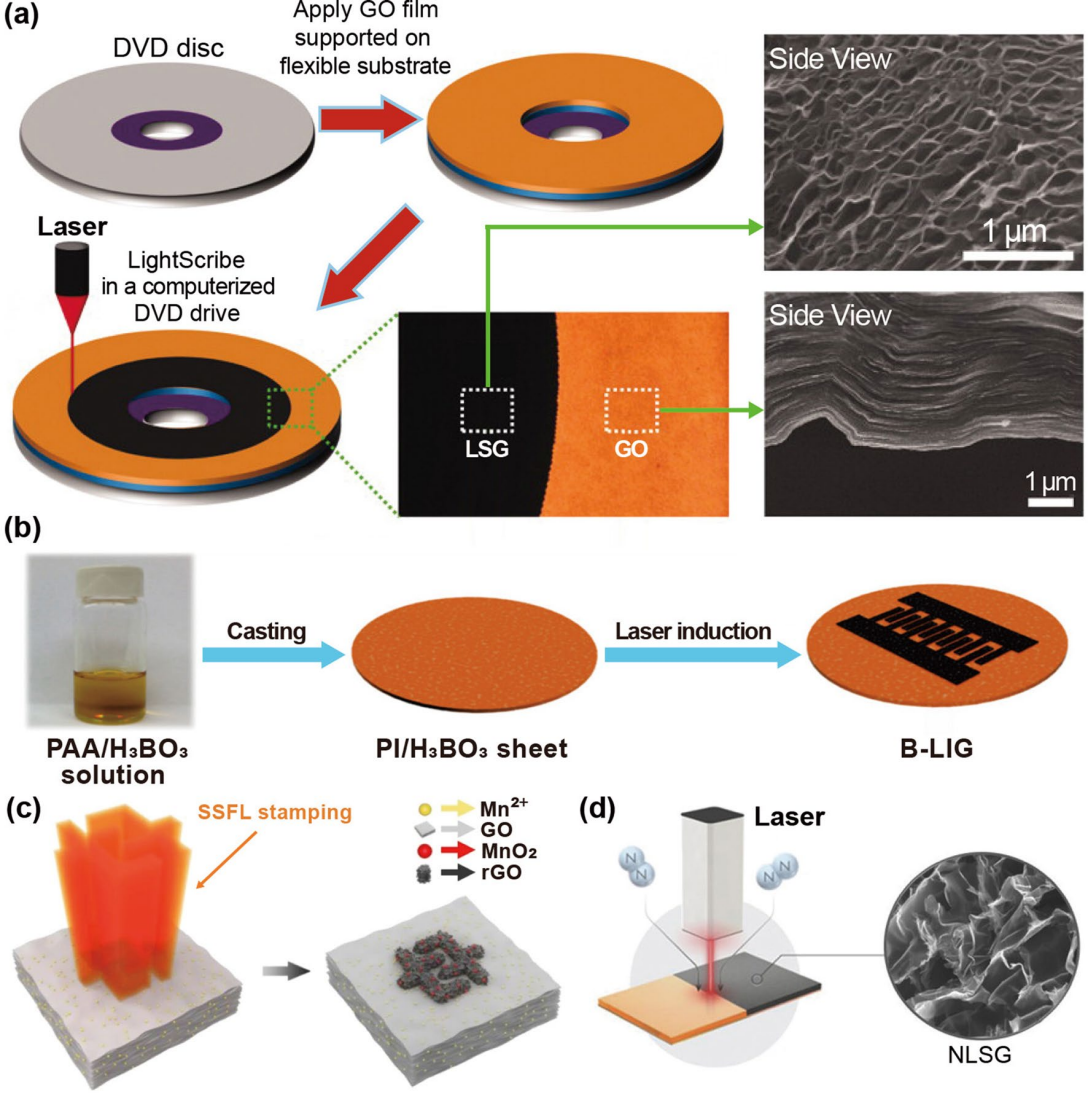
Schematic of the fabrication process for **a** laser-scribed graphene-based ECs. Reproduced with permission from Ref. [198]. Copyright 2012, The American Association for the Advancement of Science. **b** Boron-doped laser-induced graphene MSCs. Reproduced with permission from Ref. [199]. Copyright 2015, ACS. **c** Graphene/MnO_2_ MSCs via spatially shaped femtosecond laser strategy. Reproduced with permission from Ref. [200]. Copyright 2020, Springer Nature. **d** Nitrogen-doped 3D graphene electrodes on Cu foil via laser scribing. Reproduced with permission from Ref. [201]. Copyright 2018, Wiley–VCH

Electrochemical capacitors (ECs), also called supercapacitors or ultracapacitors, have been extensively investigated as energy storage devices owing to their ultrafast charge/discharge rates compared to batteries. However, they have limited energy storage densities because only the electrode surface portions can store charge. Maher et al. [198] employed light scribing to chemically induce porous graphene films to achieve high power and energy densities (Fig. 10a). Graphene oxide (GO) nanoparticles were drop-coated onto a flexible substrate, followed by infrared laser irradiation. The GO film was converted into laser-scribed graphene (LSG) via a chemical reduction reaction between the laser and GO. As shown in the cross-sectional SEM images in Fig. 10a, the stacked GO sheets were chemically reduced to well-exfoliated layered LSG films via laser irradiation. The fabricated LSG films exhibited high electrical conductivity of 1738 S m^−1^, a high specific surface area of 1520 m^2^ g^−1^, and excellent mechanical flexibility, allowing them to be utilized directly as EC electrodes without binders or current collectors, unlike traditional ECs.

Pure graphene is restricted by its inferior capacitance compared to pseudocapacitive materials. Therefore, pseudocapacitive materials (such as SnO_2_, MnO_2_, and Fe_3_O_4_) have been used as dopants to enhance the specific capacitance of graphene-based devices [202–204]. Peng et al. [199] utilized a laser induction method to fabricate boron-doped laser-induced graphene MSCs (B-LIG-MSCs) with outstanding electrochemical performance (Fig. 10b). H_3_BO_3_ (0–8 wt%) was dissolved in a solution of poly(pyromellitic dianhydride-co-4,4′-oxydianiline amic acid) (or poly(amic acid), PAA), which was cast to prepare a boric acid-containing PI film (PI/H_3_BO_3_). A CO_2_ laser was used to irradiate the as-prepared PI sheet under ambient conditions selectively. The surface of the PI sheet containing H_3_BO_3_ was transformed into B-LIG by laser irradiation. The B-LIG on the PI film was patterned into interdigitated structures to fabricate flexible MSCs, which resulted in enhanced electrochemical performance compared to non-doped LIG structures. The as-prepared devices retained their cyclability and flexibility, demonstrating the potential of B-LIG materials as next-generation, cost-effective energy storage devices.

Yuan et al. [200] employed a spatially shaped femtosecond laser (SSFL) method to fabricate LIG/MnO_2_ flexible MSC. Figure 10c shows a schematic diagram of the MSC fabrication process using the SSFL method. LIG/MnO_2_ composites were prepared by photo-modulation (photochemical and photothermal reduction or oxidation) with the advantages of a femtosecond laser, such as extremely high peak power (1013 W cm^−2^) and short irradiation time. Mn^2+^, with its high oxidation potential, was oxidized to MnO_2_ nanoparticles, assisting in the real-time reduction of GO through its anchor sites. Simultaneously, a three-dimensional fluffy porous structure with an ultrahigh-specific surface area and durability was achieved. The resulting MSC via SSFL has a high area, volumetric capabilities of 128 mF cm^−2^ and 426.7 mF cm^−3^, and an ultra-small-time response of 0.01 ms.

Moreover, a single-step direct laser scribing process was used to fabricate nitrogen-doped 3D graphene anodes on Cu foil [201]. Figure 10d presents a schematic of the nitrogen-doped 3D graphene electrodes on the Cu foil, as demonstrated by the laser scribing process. Urea-doped polyimide solutions were cast onto Cu foils and then irradiated by laser at room temperature under nitrogen flow, resulting in 3D graphene architecture with 13% nitrogen-atom doping (nitrogen-doped laser-scribed graphene, NLSG). This NLSG process was utilized to fabricate binder, conductive, and additive-free anodes with a high capacity of 425 mAh g^−1^ and outstanding cycling stability and rate performance. In addition to laser-induced photothermal and photochemical processes, laser-induced electrochemical reactions are also one important field, and laser has been successfully employed to enhance electrochemical dissolution and induce electrodeposition. Zhu et al. [205] proposed an auto-coupling mechanism between ps-laser irradiation and electrochemical atomic dissolution. Laser irradiation on the top Ge surface generates localized hot zones and mass electron–hole pairs, resulting in the formation of a highly electrical conduction channel across the Ge wafer. This channel allows current to pass preferentially and selectively under applied bias, enabling localized and enhanced electrochemical anodic dissolution. Theoretical simulation and experimental studies investigated the laser-induced electrodeposition on Si surfaces [206]. Simulation and experimental results revealed the localized enhancement of electrical conductivity via laser-induced thermal and photoconductivity effects. The laser irradiation induced a transient conductive channel inside the Si wafer through laser-induced thermal and photoconductivity effects and generated several electrons near the laser irradiation area, locally enhancing of electrical conductivity and improving electrodeposition.

### Chemical Reaction (Flash)

Flash lamp technology has emerged as a simple and large-area-processable method for developing energy materials and devices, driven by its potential to induce beneficial chemical reactions, such as reduction and doping. This technique utilizes intense, pulsed flashes of light to induce high temperatures over a short period, allowing for precise photo-thermo-chemical control of material properties without the thermal degradation commonly associated with conventional annealing methods [207–212]. This method enhances energy material performance and efficiency by activating of specific chemical reactions and forming novel material phases.

**Fig. 11:**
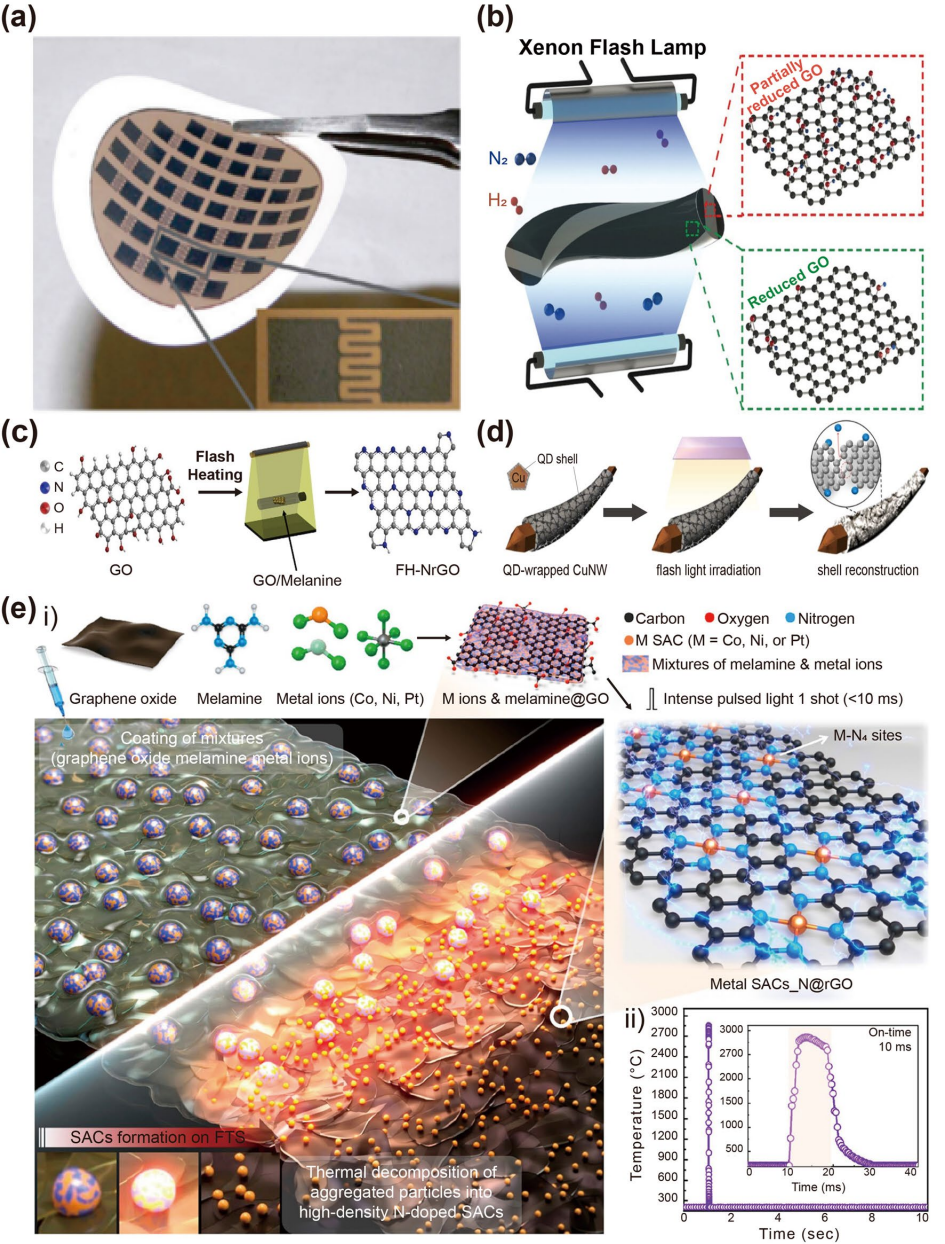
**a** Flash-induced interdigitated electrode arrays on flexible nylon film composed of reduced graphene oxide and polystyrene. Reproduced with permission from Ref. [213]. Copyright 2009, ACS. Schematic of **b** flash reduction of graphene oxide fiber. Reproduced with permission from Ref. [71]. Copyright 2019, Wiley–VCH. **c** Flash-heated nitrogen-doped reduced graphene oxide (FH-NrGO). Reproduced with permission from Ref. [214]. Copyright 2019, Elsevier. Synthesis of two-dimensional (2D) material-wrapped copper nanowires via 2D quantum dot-assembled flash light irradiation. Reproduced with permission from Ref. [215]. Copyright 2023, Elsevier. **e-i** Overall flash-thermal shock process for synthesis of single-atom catalyst-stabilized N-doped graphene; **e-ii** temperature vs. time response of metal-melamine graphene oxide. Reproduced with permission from Ref. [216]. Copyright 2023, ACS

Figure 11a shows flash-induced interdigitated electrode arrays on a flexible nylon film made of reduced graphene oxide (rGO) and polystyrene [213]. The graphene oxide blend in the polystyrene beads was chemically reduced in the air by illuminating a patterned shadow mask with a flash light, enabling the deoxygenation of graphene oxide by photothermal heating. These patterned rGO/polystyrene composite electrodes displayed a lower sheet resistance of 9.5 kΩ sq^−1^ than that of pristine GO/polystyrene (2 × 10^5^ kΩ sq^−1^), which can be used as a functional surface for energy devices.

A schematic of the flash-induced photoreduction of graphene liquid crystalline fibers (GF) is presented in Fig. 11b [71]. The graphene oxide fiber was effectively reduced in a mixture of N_2_ and H_2_ gases using a millisecond flash light with a broad wavelength spectrum. Because the reduction level can be precisely controlled through directional flash irradiation, the balance between the mechanical properties and electrical conductivity in graphene fibers can be finely adjusted to achieve specific desired characteristics. This facile, ultrafast, and customized reduction method is useful for designing materials for energy storage and conversion applications.

Although heteroatom doping of graphene can effectively improve its electromechanical properties, unstable bonding between carbon and nitrogen at high temperatures restricts nitrogen doping. Yoo et al. [214] utilized flash heating (FH) to fabricate high-concentration nitrogen-doped reduced graphene oxide (NrGO). Figure 11c schematically illustrates the FH method for demonstrating flash-heated NrGO (FH-NrGO). This FH method induces rapid heating and cooling of the graphene layers as they act as light-absorbing layers for photothermal conversion. The product graphene exhibited a corrugated structure with a large specific surface area owing to flash-induced thermal quenching. This process allows for the reduction of graphene oxide doped with a high nitrogen concentration, generating anodes with outstanding electrochemical properties for energy storage devices.

Kim et al. [215] applied flash light irradiation to synthesize two-dimensional (2D) quantum dot (QD)-wrapped Cu NWs with enhanced physical properties for transparent energy applications (Fig. 11d). A uniform thin 2D QD layer was chemically bonded to the surfaces of the Cu NW via solution processing. This QD-wrapped Cu NW was irradiated with a flash light (pulse width of 200 µs) under low-temperature and nonvacuum conditions to fabricate a highly organized and uniform shell morphology on the Cu NWs. The 2D material-wrapped Cu NWs when used as conducting electrodes for transparent supercapacitors showed outstanding oxidation stability, chemical stability, and mechanical endurance.

Moreover, an IPL-induced flash-thermal shock (FTS) lamping process was employed to prepare single-atom catalysts (SACs: Co, Ni, Pt, and Co–Ni) and nitrogen-doped graphene (Fig. 11e-i) [216]. This process enabled instantaneous soaring-temperature annealing at 2850 °C in 10 ms with ramping/cooling speeds of 105 K s^−1^ (Fig. 11e-ii), leading to nitrogen-doping of the graphene oxide substrate. The high-density active N-doping sites formed on the substrate surface facilitated the anchoring and stabilization of the SACs at high capacities. Melamine was used as an N-dopant to form metal–nitrogen bonding sites by thermal heating, generating a uniform, high-density atomic distribution of single metal atoms.

#### Synthesis (Laser + Flash)

Laser- and flash-induced technologies with non-equilibrium photon interaction characteristics have been utilized to synthesize materials with enhanced functionalities for advanced energy conversion and storage applications. LMI-derived synthesis processes use substantial amounts of reactants to create entirely different types of compounds by forming new bonds between atoms or groups of atoms, which can be scaled up depending on the desired quantity of the end product. These technologies can engineer physical/chemical interactions and material structures via precise spatiotemporal energy input. By initiating and controlling photo-thermo-chemical reactions via an intense and instantaneous LMIs, materials such as quantum dots, metal oxides, and complex hybrids can be synthesized. These materials with unique optoelectronic, piezoelectric, and electrochemical properties are crucial for innovative energy conversion and storage technologies, such as high-performance energy harvesters, supercapacitors, and batteries.

**Fig. 12:**
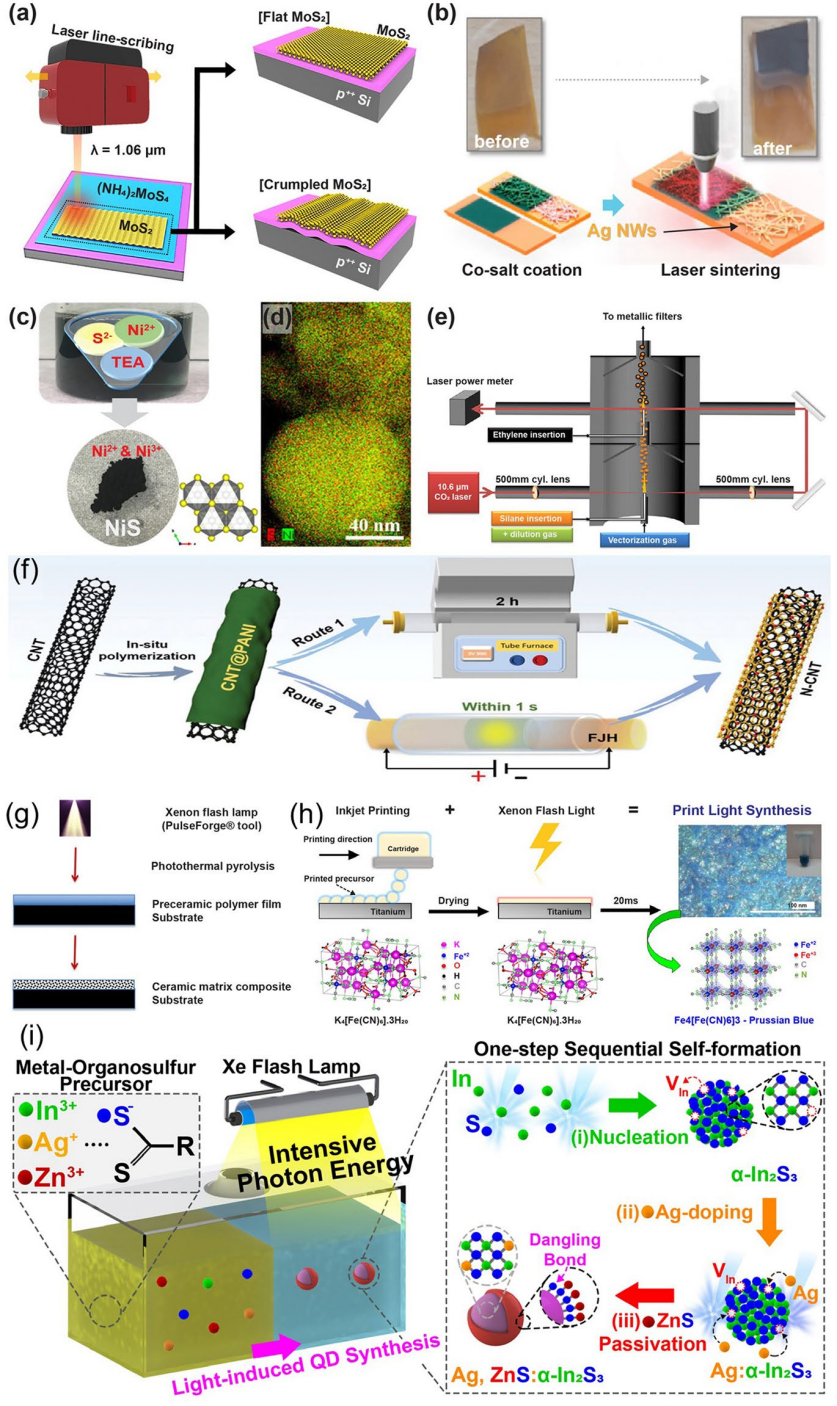
**a** Schematic of laser-directed synthesis of 2D MoS_2_ on SiO_2_/Si substrate. Reproduced with permission from Ref. [83]. Copyright 2020, Elsevier. **b** Synthesis process of laser-sintered carbon-cobalt oxide composites. Reproduced with permission from Ref. [217]. Copyright 2023, Elsevier. **c, d** Schematic of laser-induced NiS nanostructures and their EDX elemental mapping. Reproduced with permission from Ref. [218]. Copyright 2019, Elsevier. **e** Schematic illustration of two-stage laser pyrolysis reactor. Reproduced with permission from Ref. [219]. Copyright 2015, ACS. **f** Schematic of conventional pyrolysis and flash Joule heating methods of the N-CNTs. Reproduced with permission from Ref. [220]. Copyright 2023, Wiley–VCH. **g** Schematic of photothermal pyrolysis of organosilicon PCP films. Reproduced with permission from Ref. [113]. Copyright 2021, ACS. **h** Schematic of overall PLS process for PB films. Reproduced with permission from Ref. [221]. Copyright 2020, ACS. **i** Schematic for flash-induced one-step sequential self-formation of metastable Ag, ZnS:α-In_2_S_3_ QDs. Reproduced with permission from Ref. [77]. Copyright 2021, Elsevier

To improve the triboelectrification of haptic sensors, Park et al. [83] employed a direct single-step laser processing method to synthesize thin, 2D MoS_2_ layers using photonic thermolysis. Figure 12a shows a schematic of the laser-directed synthesis of 2D MoS_2_ on a SiO_2_/Si substrate. The patternable laser synthesis successfully controlled the MoS_2_ crystal surface morphology by inducing internal strain, enabling the fabrication of flat and crumple MoS_2_ structured TENG devices in a nonvacuum atmosphere without any treatment/modification.

Transition metal oxides combined with carbon materials are receiving considerable attention for enhancing the electrical conductivity and electrochemical properties of energy storage devices. However, their synthesis requires numerous toxic chemicals and a long calcination process that releases harmful gases. Duy et al. [217] utilized laser sintering to synthesize carbon and cobalt oxide (Co_3_O_4_) composites (C-CoO_x_) to fabricate flexible supercapacitors. Figure 12b shows the synthesis process of the laser-sintering-induced carbon–cobalt oxide composites. This composite was well crystallized and firmly attached to an Ag NW-coated PI substrate via low-power (1 W) laser irradiation, demonstrating a flexible supercapacitor with excellent electrochemical performance.

Similarly, Hung et al. [218] reported the synthesis of nickel sulfide (NiS) nanostructures by laser irradiation of an aqueous solution under ambient conditions without calcination. Figure 12c, d shows the laser-induced NiS nanostructure and its energy-dispersive spectroscopy (EDS) elemental mapping results, respectively. The LMI-assisted NiS enabled better crystallinity and high phase purity with outstanding physical properties, such as electrochemical performance, specific capacitance, and rate capability for supercapacitor devices. Wang et al. [222] utilized a laser-induced transient self-organization technique to prepare hierarchical pulsed laser-induced TiN_x_ (LITN) nano-filament networks by regulating the thermodynamic and kinetic processes. Laser-induced TiN-based surface mountable filter capacitors exhibit regular 3D hierarchical network structures made of ultrafine nano-filaments with a diameter of 3–5 nm and high energy density of 9.17 mWh cm^−3^ at 120 Hz, significantly higher than that of nitrides and carbon-based supercapacitor electrodes used in high-frequency applications.

To realize high-capacity anode materials for LIBs, Si/C core–shell nanoparticles (Si@C) were synthesized by laser-driven chemical vapor pyrolysis (LCVP) in two stages [219]. Figure 12e shows a schematic diagram of the two-stage laser pyrolysis reactor. The laser beam initially interacted with the silane gas in the bottom stage, leading to the formation of crystalline silicon cores. Subsequently, the carrier gas rapidly transported the silicon cores to the upper stage, where carbon shells were deposited onto these cores.

In recent years, nitrogen-doped carbon nanomaterials have received great attention in electrochemical energy conversion and storage applications. Zhang et al. [220] utilized a flash Joule heating method to enhance energy storage performance and cycle stability of nitrogen-doped carbon nanotubes (N-CNTs). This method first enabled a 1D core–shell structure of CNT@polyaniline in-situ polymerization process, and further, it converted into nitrogen-doped carbon layer on the surface of nanotube by two heating techniques, such as conventional pyrolysis and flash Joule heating, leading to the formation of flash N-CNT, as presented in Fig. 12f. These outstanding electrochemical responses via the Joule heating method for preparing heteroatom-doped carbon nanomaterials are promising for energy conversion and storage applications.

Uzodinma et al. [113] synthesized silicon carbide (SiC) and silicon oxycarbide (SiOC) ceramic/carbon nanocomposites through the flash photothermal pyrolysis of organosilicon preceramic polymers (PCPs) for electrochemical energy storage applications. Figure 12g shows a schematic of the flash lamp processing procedure for the photothermal pyrolysis of organosilicon PCP films. Upon exposure to flash light, the organosilicon was transformed into a carbon/ceramic nanocomposite without thermal deformation of the heat-sensitive substrate. The flash-induced nanocomposite film exhibited a high capacitance of 27.2 mF cm^−2^, better stability over 1000 cycles, and a high coulombic efficiency of 80%, showing potential for energy storage and catalysis.

Silva et al. [221] utilized an oxidative print light synthesis (PLS) method to fabricate Prussian blue (PB) films with improved electrocatalytic performance. Figure 12h shows a schematic of the overall PLS process for the PB films. Potassium hexacyanoferrate (II) trihydrate precursor (K_4_[Fe(CN)_6_]·3H_2_O) ink with Nafion solution (5 wt%) was deposited on a titanium substrate via inkjet printing. The printed K_4_[Fe(CN)_6_]·3H_2_O film was dried and sintered in 20 ms via flash light irradiation, forming Fe_4_[Fe(CN)_6_]_3_ with a blue color. The PLS PB film exhibited an outstanding electrocatalytic performance during the charge/discharge processes, which is promising for energy-storage device applications such as LIBs.

Moreover, metastable QDs have been synthesized by flash light-induced sequential self-formation for applications in photoelectric devices [77]. The multiple irradiations of flash pulses enabled α-In_2_S_3_ QD nucleation, Ag doping, and ZnS passivation by photoresponsive ionic kinetics (Fig. 12i). The as-synthesized QDs were utilized to fabricate a metal–semiconductor-metal photoelectric device with excellent optoelectronic performance and long-term stability.

The complexity of the light-induced synthesis process stems from its dependence on highly specific and non-equilibrium LMI conditions, characterized by ultrafast interactions, and LMI parameters with high degrees of freedom. This feature limits the establishment of theoretical LMI-induced synthesis mechanisms for identifying optimized light processing conditions. One of the promising solutions lies in leveraging computational power to integrate and analyze data from multiscale simulations and feedback experimental results. This integrative approach aims to use machine learning and artificial intelligence to develop robust models that can predict the outcomes of LMI-driven synthesis processes with high accuracy, reliability, and efficiency. Table 2 shows summarization of light sources of LMI technique, applications, and other details, applied for light-induced energy conversion and storage field.

**Table 2:** Summarization table of light sources, applications, and other details, applied for light-induced energy conversion and storage applications

	Laser	Wavelength	Interaction time	Process	Applications	References
Femtosecond/picosecond	Ti:sapphire	800 nm	30 fs	Texturing	Laser-induced light absorbers	[81]
	Yb:YAG	1030 nm	1 ps, 20 µm diameter	Synthesis	Laser-induced synthesis of perovskite	[82]
	Fiber laser	1.06 μm	100 ps	Synthesis	Laser-induced synthesis of MoS_2_	[83]
Excimer	XeCl	308 nm	30 ns	LLO	Energy harvester (PZT film)	[89]
	XeCl	308 nm	30 ns	LLO	Energy harvester (TE generator)	[116]
	XeCl	308 nm	30 ns	LLO	PZT film energy harvester	[37]
	XeCl	308 nm	30 ns	LLO	Piezoelectric sensor	[223]
	XeCl	308 nm	30 ns	LLO	Piezoelectric pressure sensor	[128]
	XeCl	308 nm	30 ns	LLO	Piezoelectric acoustic sensor	[224]
	XeCl	308 nm	30 ns	LLO	Ceramic capacitor	[119]
Flash lamp	Flash lamp	200—1100 nm	0.2 ms	LLO	Solar cell	[103]
	Flash lamp	350—1500 nm	0.25 to 1 ms	Crystallization	PZT film	[115]
	Flash lamp	200—1100 nm	0.3 ms	Crystallization	Magnetoelectric composites	[144]
	Flash lamp	200—1100 nm	0.66 ms	Welding	Ag NW	[114]
	Flash lamp	190—1000 nm	0.5 ms	Sintering	Polymer solar cell	[92]
	Flash lamp	200—1100 nm	0.1 to 1 ms	Sintering	Si anode for LIB	[93]
	Flash lamp	200—1100 nm	3 ms	Texturing	Magneto-mechano-triboelectric nanogenerator	[94]
	Flash lamp	200—1100 nm	0.8 ms	Pyrolysis	Graphene films on arbitrary substrates	[95]
	Flash lamp			Reaction	Supercapacitor	[225]
	Flash lamp	190 nm to 800 nm	1–2 ms	Reaction	Patterning graphene oxide	[96]
	Flash lamp		1 ms	Synthesis	Photothermal pyrolysis	[113]
Continuous wave	Nd:YAG	532 nm	20 μm, 0.1 mm s^−1^ (0.2 s)	Annealing	PZT film	[104]
	CO2 laser	10.6 μm	pulse duration: 14 μs, 3.5 in s^−1^	Texturing	Fluorinated polyimide films	[102]
	CO2 laser	10.6 μm	pulse duration: 14 μs, spot size of 120 μm	Pyrolysis	Graphene films on polymers	[99]

## Applications of LMIs in Energy Conversion and Energy Storage Devices

LMI processes, characterized by their instantaneous, spatially selective, multiphysical, and nonequilibrium photon engagement, have been instrumental in enabling unique photothermal and photochemical reactions across a variety of materials, including ceramics, metals, polymers, semiconductors, and carbon-based substances [68, 70]. The extensive application of LMI technologies in energy conversion and storage has led to significant advancements in various devices [89, 119, 128]. These devices include energy harvesters, sensors, capacitors, and batteries, making LMI technology pivotal for future electronic systems centered on energy conversion and storage. In this section, we explore cutting-edge applications in energy-harvesting systems, mechanical/magnetic sensors, and energy-storage devices such as capacitors and batteries. These applications demonstrate the innovative utilization of LMI techniques, including photothermal reactions, light-induced annealing, and photochemical responses. Table 3 lists the primary processing parameters for the LMI process used in energy conversion and storage device applications introduced in this section.

**Table 3:** Processing parameters for LMIs in energy conversion and storage applications

Energy conversion and storage applications	LMI method	Process Parameters	References
Flexible PZT-based energy harvester, sensor, and capacitor	LLO (Laser)	Wavelength: 308 nm	[37, 119, 128, 223, 226]
		Fluence: 0.42 or 0.61 J cm^−2^	
		Pulse duration: 30 ns	
		Environment: air	
Ag NW-based transparent flexible harvester	Plasmonic welding (Flash light)	Wavelength: 300–1000 nm	[114]
		Fluence: 10.3 J cm^−2^	
		Pulse duration: 0.66 ms	
		Environment: air	
MMTENG for optogenetic neuromodulation	Surface annealing (Flash light)	Wavelength: 300–1000 nm	[94]
		Fluence: 20 J cm^−2^	
		Pulse duration: 3 ms	
		Environment: air	
PZT/Metglas ME heterostructure	Crystallization (Flash light)	Wavelength: 350–1500 nm	[115]
		Fluence: 5.6 J cm^−2^	
		Pulse duration: 0.75 ms	
		Environment: air	
Flexible thermoelectric generator	LLO (Laser)	Wavelength: 308 nm	[116]
		Fluence: 0.7 J cm^−2^	
		Pulse duration: 30 ns	
		Environment: air	
Roll-to-roll inkjet Ag grid-based solar cell	Sintering (Flash light)	Wavelength: 190–1000 nm	[92]
		Flash energy: 830 J	
		Pulse duration: 0.5 ms	
		Environment: air	
Crumpled MoS_2_ TENG device	Thermolysis (Laser)	Wavelength: 1060 nm	[83]
		Fluence: 2.52–2.62 J cm^−2^	
		Pulse duration: 100 ps	
		Environment: air	
Magnetic field sensor using ME composite	Crystallization (Flash light)	Wavelength: 300–1000 nm	[144]
		Fluence: 7.1 J cm^−2^	
		Pulse duration: 0.3 ms	
		Environment: air	
Microsupercapacitive stone module	Reduction sintering (Laser)	Wavelength: 1070 nm	[98]
		Laser power: 10 W	
		Pulse duration: Not marked	
		Environment: air	
Silicon anode-based lithium-ion battery	Annealing, surface modification (Flash light)	Wavelength: 350–1500 nm	[93]
		Fluence: 0.75, 1.9 J cm^−2^	
		Pulse duration: 1, 0.1 ms	
		Environment: air	
TiN_x_ nano filament percolated capacitor	Heating (Laser)	Wavelength: 1064 nm	[222]
		Laser power: 13 W	
		Pulse duration: 2–350 ns	
		Environment: N_2_ gas	

### LMI Process for Energy Harvesting Devices

**Fig. 13:**
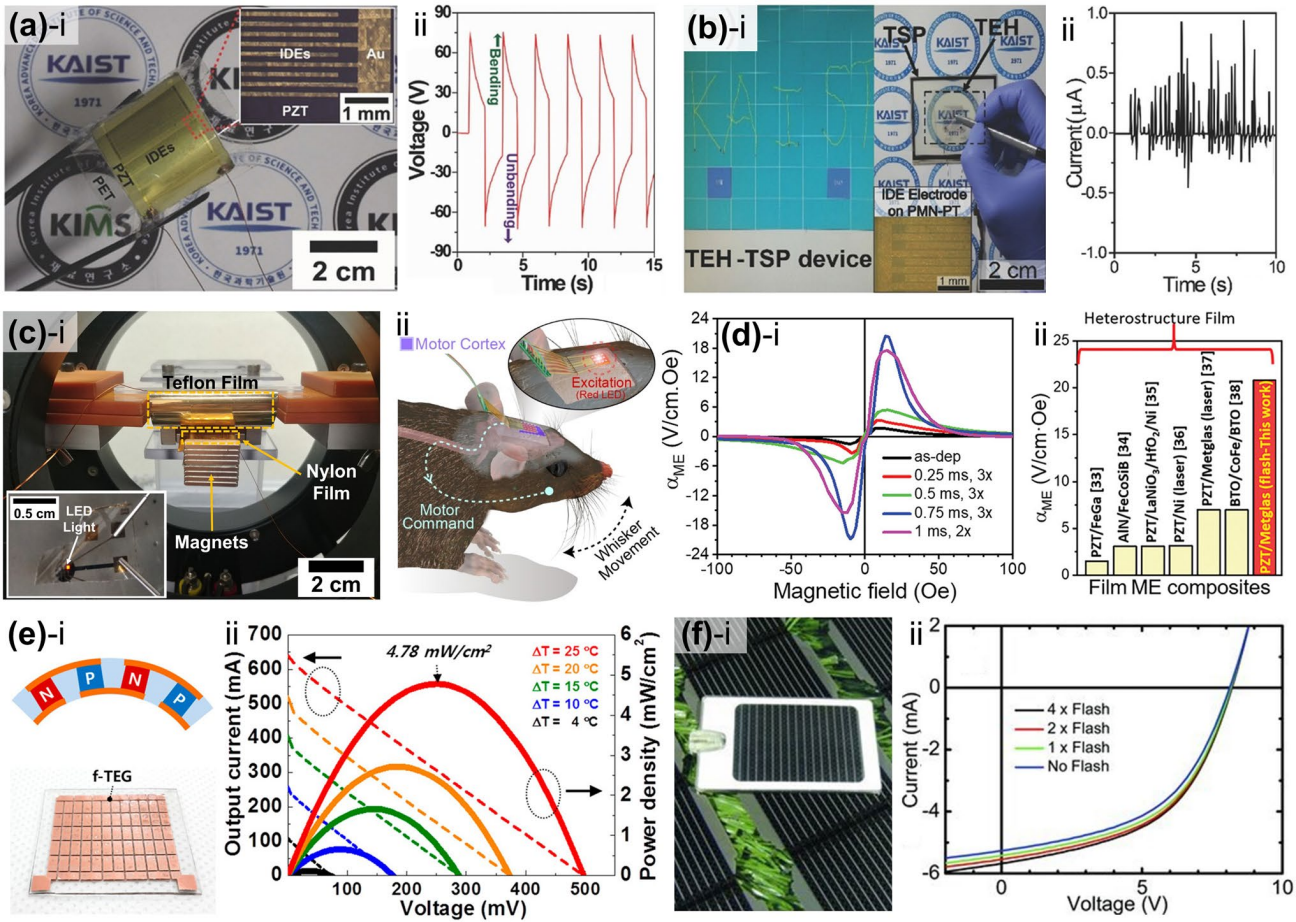
**a-i** Photographic depiction of LLO-based flexible PZT energy harvester; inset: optical microscopy image of the harvester top electrode layer; **a-ii **Graph representation of open-circuit output voltage measurements obtained from flexible PZT energy harvester. Reproduced with permission from Ref. [37]. Copyright 2016, Wiley–VCH. **b-i** Image capturing TSP energy harvesting system integrated with a flexible, transparent AgNWs electrode layer; inset: microscopic visualization of AgNWs electrode layer on piezoelectric material; **b-ii** graph delineating short-circuit current response of TSP harvester during handwriting motions. Reproduced with permission from Ref. [114]. Copyright 2017, Wiley–VCH. **c-i** Photographic portrayal of MMTENG device positioned within a Helmholtz coil; inset: illustration of f-μLED powered by the MMTENG setup. **c-ii** schematic representation of self-powered optogenetic brain stimulation experiment conducted on mouse model. Reproduced with permission from Ref. [94]. Copyright 2020, Elsevier. **d-i** Comparative analysis graph of α_ME_ values observed in pristine PZT vs. various PZT flash irradiation conditions applied on Metglas substrate. **d-ii** comparative graph showcasing α_ME_ values of flash-induced PZT/Metglas bilayer in relation to previously reported ME heterostructures. Reproduced with permission from Ref. [104]. Copyright 2023, Wiley–VCH. **e-i** Photographic image alongside a cross-sectional view detailing the thermoelectric structure of the f-TEG device. **e-ii** graph illustrating measured output characteristics of f-TEG under varying ΔT conditions. Reproduced with permission from Ref. [116]. Copyright 2016, American Chemical Society. **f-i** Photographic representation of a solar cell module. **f-ii** IV curve graph displaying solar module performance using pristine and various flash sintering-conditioned samples. Reproduced with permission from Ref. [92]. Copyright 2013, Wiley–VCH

Figure 13a-i displays a flexible piezoelectric energy harvester utilizing the LLO process. The XeCl excimer laser pulses (pulse duration of 30 ns) irradiated to the PZT through the transparent substrate caused melting of PZT/substrate interface, successfully reducing the adhesion of PZT film. This technique facilitated the transfer of a high-temperature (900 °C) annealed piezoelectric PZT thick film (7 μm in thickness) from a rigid sapphire substrate to a plastic film while preserving both structural and chemical integrity [37]. The resulting LLO flexible harvester demonstrated substantial electrical outputs, generating open-circuit voltages up to 75 V (as seen in Fig. 13a-ii) and short-circuit currents of 14 μA through bending and releasing motions. In addition, they employed a flexible PZT harvester to charge a 1 mF capacitor up to 4.3 V and used the capacitor to operate a wireless temperature sensor for illustration of its application potential. This setup successfully utilized the stored energy for the measurement of the ambient temperature, achieving 18 consecutive operations along with simultaneous data transmission to a transceiver.

Figure 13b-i introduces a novel touchscreen-based energy harvesting system that integrates a transparent, flexible piezoelectric harvester with a touchscreen panel (TSP) [114]. This system employs AgNWs as the electrode layer in transparent harvesting devices. Owing to the broad spectrum of flash light, randomly arranged Ag NWs on the flexible piezoelectric film were effectively welded via the plasmonic heating effect, resulting in a low sheet resistance at high transparency. The incorporation of AgNWs as the electrode layer, welded through flash light-induced plasmonic interaction, enabling low sheet resistance and high transparency of the piezoelectric material. A transparent piezoelectric harvester with a transparency of 80% was capable of generating significant electrical outputs, demonstrated by the open-circuit voltage of 38 V and a short-circuit current of 12.5 μA. Additionally, when integrated with TSP, it was able to produce an oscillating output of 20 V and 1 µA under pen-writing motions, as depicted in Figure 13b-ii.

Figure 13c-i describes the development of a flash-enhanced MMTENG designed for optogenetic neuromodulation applications by powering in vivo flexible μLEDs (f-μLEDs) [94]. Nanoscale bumpy textures and microscale wrinkle structures were concurrently developed at a CuO/Nylon interface by exposing a millisecond flash light onto the CuO NPs coated Nylon film. After chemically etching the flash-derived Cu structures, the Nylon film surface with enhanced active friction area could be achieved. The MMTENG device with a flash-induced nylon surface showed a 2.6-fold increase in output power compared with the pristine polymer material. This enhancement enabled the activation of f-μLEDs by converting ambient magnetic field noise into electricity, as depicted in the inset of Fig. 13c-i. A schematic representation in Fig. 13c-ii illustrates a self-powered optogenetic brain stimulation setup that involves a living mouse, an array of f-μLEDs powered by the MMTENG, and a motor command pathway that triggers whisker movement. The in-vivo energy-scavenged f-μLEDs optogenetically activated the Chrimson-modified primary motor cortex in the mouse. This activation induced the movement of mouse whiskers, demonstrating the practical application of MMTENG in a biological context.

Figure 13d-i shows the ME voltage coefficient (α_ME_) graphs of the aerosol-deposited (AD) PZT/FeBsi (Metglas) heterostructure films [115]. The flash treatment achieved selective crystallization of the target PZT thick-film layer without thermal damage to the Metglas foil. The optimal flash light conditions, three consecutive shots with a pulse duration of 0.75 ms, yielded a maximum α_ME_ value of 20.46 V cm^−1^ Oe^−1^. This value represents a notable advancement in magnetoelectric conversion performance, surpassing that of previously reported heterostructured ME film composites, as illustrated in Fig. 13d-ii. The enhanced performance can be attributed to the significant polarization effect in the flash-induced PZT film coupled with the optimal interfacial interactions within the PZT/Metglas heterostructured ME film composite.

Figure 13e-i illustrates an f-TEG fabricated through the screen printing of active thermoelectric films (p-type Bi_0.3_Sb_1.7_Te_3_ and n-type Bi_2_Se_0.3_Te_2.7_) onto a quartz substrate [116]. This process was followed by multi-scanning LLO technique to attain a freestanding device state. Successful exfoliation of these films was achieved by generating Si nanoparticles within the amorphous Si layer using high-energy excimer laser irradiation. This approach effectively minimized the surface contact area between the active thermoelectric materials and the parent substrate. The f-TEG module, as depicted in Fig. 13e-ii, not only demonstrates a high output power density of 4.78 mW cm^−2^ at a temperature differential (ΔT) of 25 °C but also exhibits remarkable flexibility and stability, maintaining functionality with a bending radius as low as 5 mm.

Figure 13f-i depicts a polymer solar cell module featuring a roll-to-roll flash photon-sintered silver grid, which serves as a semitransparent front electrode layer for organic solar cells [92]. The Ag NPs patterned onto a flexible PET film by inkjet printing were sintered by irradiating the flash light, resulting in the decrease of the sheet resistance through the grain growth and densification of the Ag NPs. This flash-induced Ag grid improved fill factor and overall performance of the solar cells compared with traditional ITO-based devices while also offering benefits in terms of ease of processing under atmospheric pressure. A detailed analysis of the relationship between the current and voltage (*I–V*) performance of solar cells and the extent of photonic sintering of the grid revealed a significant trend: an increase in the number of flash shots correlated with an enhanced photocurrent. Additionally, there were slight, yet noticeable, improvements in both the fill factor and open-circuit voltage of the device after flashlight treatment.

### LMI Process for Mechanical/Magnetic Sensing Devices

**Fig. 14:**
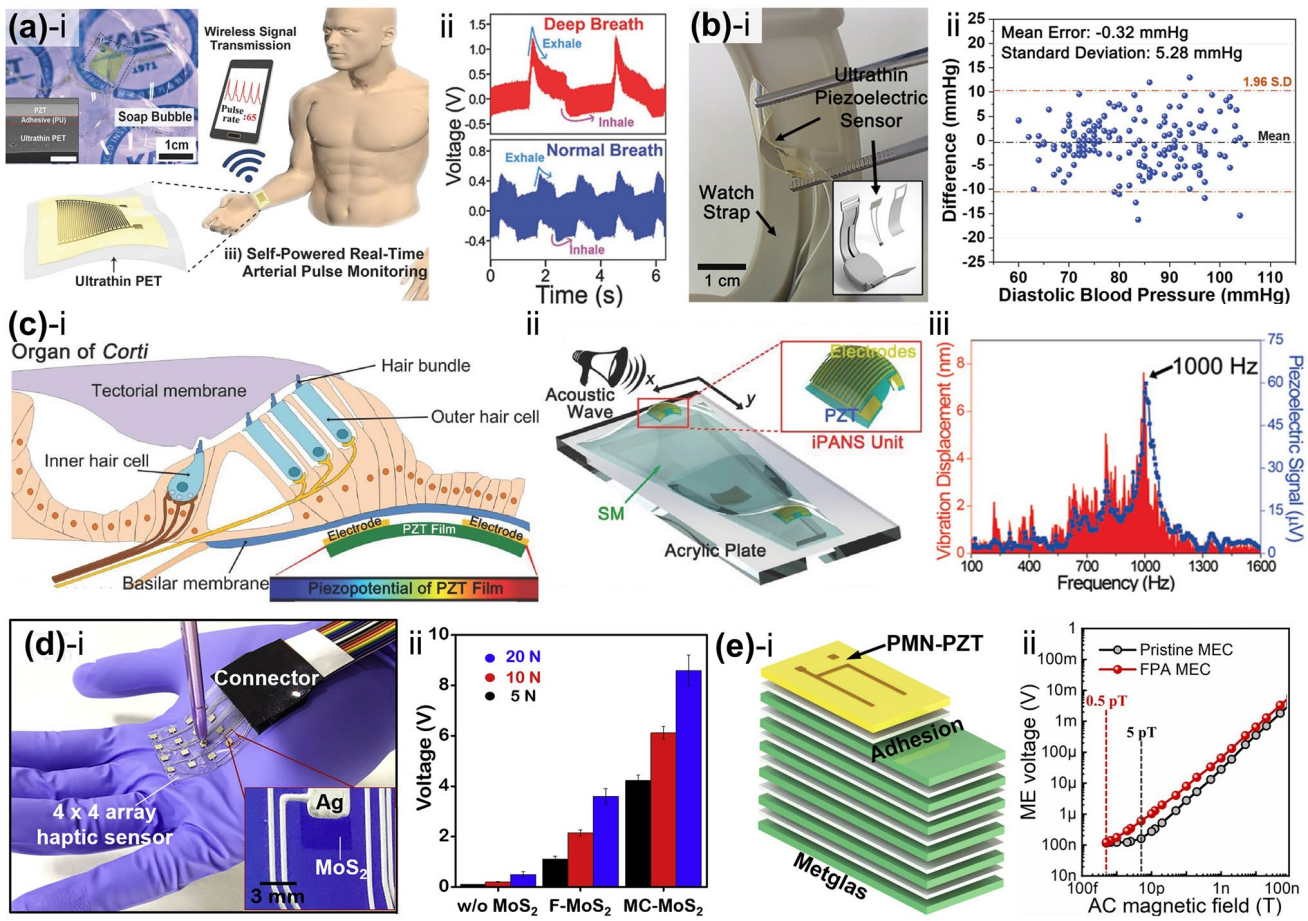
**a-i** Schematic representation of a self-powered piezoelectric pressure sensor designed for wireless monitoring of arterial pulse at human wrist; inset shows a photographic depiction of an ultrathin flexible PZT sensor demonstrating buoyancy on soap bubbles. **a-ii** Graph showcasing output voltage signals from piezoelectric pressure sensor during normal and intensified oral breathing. Reproduced with permission from Ref. [223]. Copyright 2017, Wiley–VCH. **b-i** Photographic image of ultrathin piezoelectric sensor integrated within inner surface of wristwatch strap; inset: conceptual design of a wristwatch-based WPBPS. **b-ii** Bland–Altman plots employed to validate WPBPS accuracy in measuring DBP, compared against readings from an oscillometric sphygmomanometer across a sample of 35 participants. Reproduced with permission from Ref. [128]. Copyright 2023, Wiley–VCH. **c-i** Conceptual schematic illustrating implementation of iPANS designed for acoustic wave detection within mammalian cochlea Organ of Corti. **c-ii** Graphic illustration depicting operational mechanism of iPANS, focusing on resonance-induced mechanical vibration of flexible PZT in response to acoustic waves. **c-iii** Illustration of vibrational displacement and subsequent piezoelectric voltage generation by iPANS located on silicone membrane structure. Reproduced with permission from Ref. [226]. Copyright 2014, Wiley–VCH. **d-i** Photograph showcasing a flexible MoS_2_-based triboelectric haptic sensor array applied to a human hand; inset: laser-synthesized MoS_2_ and Ag electrode lines in the haptic sensor. **d-ii** Graph presenting voltage output signals from the triboelectric haptic sensor, comparing scenarios without the MoS_2_ layer and with flat or crumpled MoS_2_ layers under varying external mechanical forces. Reproduced with permission from Ref. [83]. Copyright 2020, Elsevier. **e-i** Schematic delineation of an ME composite magnetic field sensor comprising a magnetostrictive Metglas lamination coupled with a piezoelectric PMN-PZT layer. **e-ii** Voltage response of the ME sensor to AC magnetic fields as low as 0.5 pT, using pristine and flash-induced Metglas sheets. Reproduced with permission from Ref. [144]. Copyright 2021, Elsevier

Figure 14a-i delineates the structure of a self-powered flexible pressure sensor on an ultrathin PET substrate (4.8 μm thick), achieved through an LLO process [223]. This process facilitates the transfer of a high-temperature-annealed PZT thin film (thickness of 2 μm) from a rigid sapphire wafer. The ultra-flexibility of the PZT film on plastic, which is capable of conformally floating on soap bubbles owing to its low bending rigidity, is illustrated in the inset of Fig. 14a-i. This flexible PZT sensor, with a sensitivity of approximately 0.018 kPa^−1^ and a response time of approximately 60 ms, can be employed for the real-time monitoring of human respiratory activities. This application was demonstrated by attaching an ultrathin sensor to a conventional medical mask to track exhalation and inhalation patterns, as shown in Fig. 14a-ii. The photographs in Fig. 14b-i show an LLO-based wearable piezoelectric blood pressure sensor (WPBPS) built into a wristwatch strap [128]. The sensor comprises a 2 μm PZT thin film on a 75 μm PET substrate with biocompatible PDMS encapsulation, ensuring conformal contact with human skin for enhanced pulse detection from the radial artery. The WPBPS exhibited high sensitivity (0.062 kPa^−1^), rapid response time (23 ms), and remarkable mechanical stability over 50,000 push cycles. Figure 14b-ii shows a comparison of diastolic blood pressure (DBP) measurements from the WPBPS and the U.S. Food and Drug Administration (FDA)-approved sphygmomanometer across 35 participants. The findings indicated a mean error and standard deviation of − 0.32 ± 5.28 mmHg for DBP when comparing our sensor with the conventional sphygmomanometer. These results confirmed the accuracy of the WPBPS in clinical blood pressure measurements across diverse demographic groups of different sexes and age ranges.

Figure 14c-i portrays an implanted flexible inorganic PZT acoustic nanosensor (iPANS), fabricated using an LLO process and strategically positioned beneath the basilar membrane in the organ of Corti to respond to auditory stimuli [226]. This development addresses the issue of permanent hearing impairment caused by the loss or damage of hair cells, which cease to generate bioelectrical potential under such conditions [227]. The use of flexible piezoelectric films as artificial hair cells has emerged as a promising alternative for restoring the functionality of impaired hair cells. Figure 14c-ii shows the acoustic frequency separator comprising a flexible PZT film, trapezoidal freestanding silicone membrane, and an acrylic frame. Upon exposure to acoustic waves, the silicone membrane undergoes vibrational motion owing to the resonance effects, which mechanically deform the iPANS on the silicone membrane, leading to the generation of piezoelectric-induced electrical signals. To assess the frequency-dependent response of the iPANS to sound stimulation, the device was subjected to sound waves in the range of 100–1600 Hz. As shown in Fig. 14c-iii, the iPANS located at the end region of the silicone membrane exhibited an obvious response to sound frequencies near 1000 Hz. This observation was attributed to the resonance frequency of iPANS, thereby validating its effectiveness as a frequency separator and selective responsiveness to specific acoustic frequencies.

Figure 14d-i illustrates a self-powered flexible touch sensor array that leverages the triboelectric effect using a 2D MoS_2_ film synthesized via laser scribing [83]. The insets of Fig. 14d-i show the establishment of Ag electrode lines on the MoS_2_ pattern achieved through inkjet printing. The pivotal aspect of this design lies in the laser-directed thermolysis of the MoS_2_ layer, which allows the manipulation of the surface morphology between flat and crumpled structures by varying the irradiated laser power. This morphological tuning enhanced the output voltage signal of the triboelectric sensor, which was attributed to the increased contact area of the crumpled MoS_2_ structure. To rigorously evaluate the triboelectric sensitivity of this flexible sensing device, especially with the most crumpled-surface MoS_2_, comparative control tests were conducted. These tests involved the use of sensors with flat MoS_2_ surfaces and those comprising only Ag electrode layers as triboelectric materials. The results depicted in Fig. 14d-iii clearly indicate that the crumpled MoS_2_ sensor generates a higher output voltage than its flat MoS_2_ and electrode-only counterparts. This superior performance was consistent across all the applied force levels of 5, 10, and 20 N, thereby underscoring the efficacy of the crumpled MoS_2_ in enhancing the triboelectric response of the sensor array.

Figure 14e-i delineates the ME laminate composite structure of an ultrasensitive AC magnetic field sensor [144]. This sensor consists of a flash photon-annealed magnetostrictive Metglas lamination and a piezoelectric Pb(Mg_1/3_Nb_2/3_)O_3_–Pb(Zr,Ti)O_3_ (PMN-PZT) crystal. This process induced nanocrystals on the Metglas surface, resulting in a 27.3% improvement in the piezomagnetic constant, 40% increase in the mechanical quality factor, and 46% enhancement in α_ME_ at the resonance frequency, as compared to the unprocessed Metglas. Furthermore, the ME responses of these sensors to the applied AC magnetic fields under resonant conditions are shown in Fig. 14r-ii. The induced ME voltages exhibited a nearly linear relationship, even at an ultralow AC magnetic field strength of approximately 1 pT. The flash-induced sensor demonstrated an exceptional magnetic field detection limit of 0.5 pT at a resonance frequency of 99.3 Hz. This performance was significantly superior by an order of magnitude compared to that of the pristine sensor with a detection limit of 5 pT at a resonance frequency of 97.7 Hz.

### LMI Process for Energy Storage Devices

**Fig. 15:**
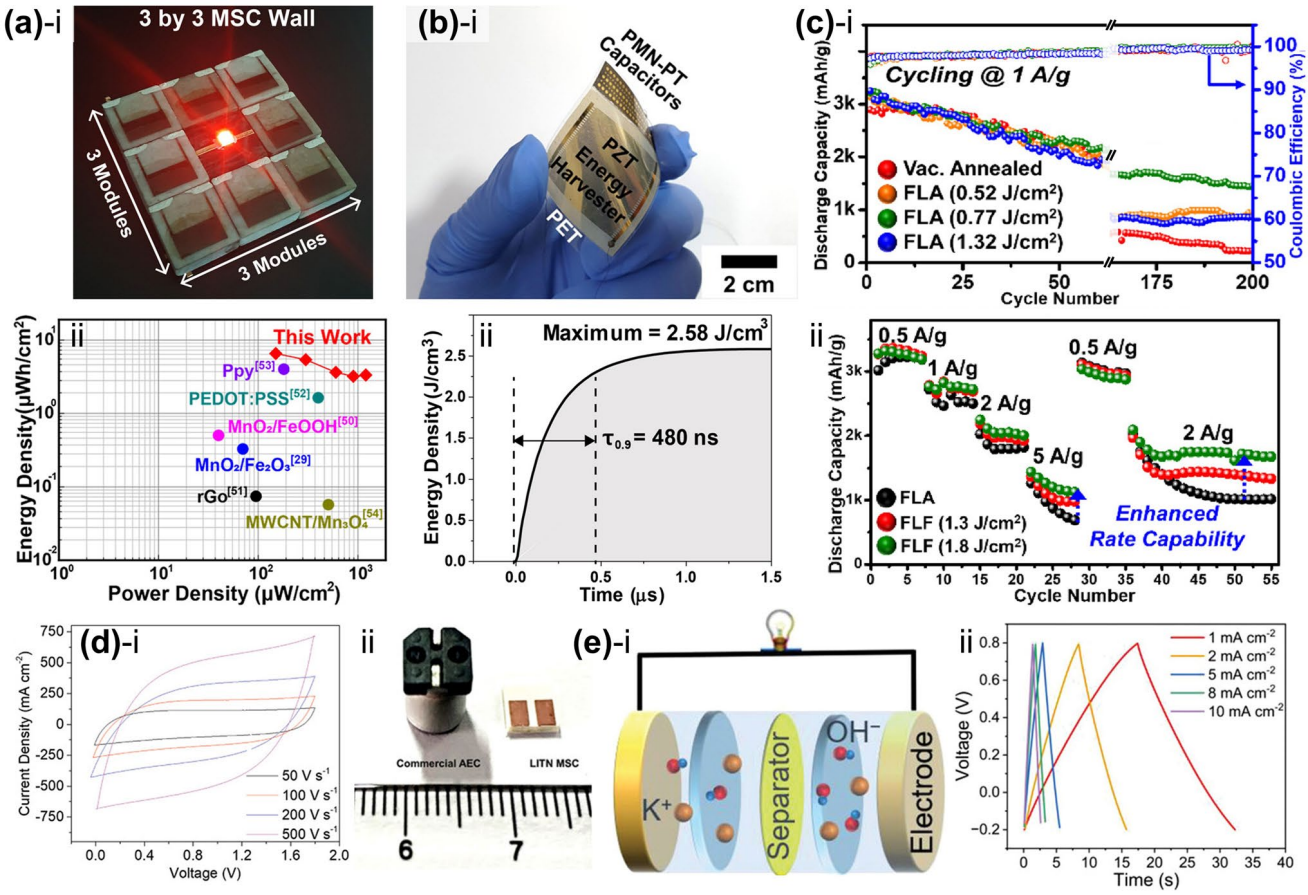
**a-i** Photograph of an LED powered by a 3 × 3 array of stone-based MSC modules; **a-ii** Ragone plot comparing the energy and power densities of the stone-based MSC module in this study with previously reported MSC devices. Reproduced with permission from Ref. [98]. Copyright 2022, American Chemical Society. **b-i** Image of the self-charging flexible ceramic capacitor system, demonstrating flexibility by bending with human fingers. **b-ii** Graph showing the discharged energy density of the self-charging flexible capacitor system under the bending motion of human fingers. Reproduced with permission from Ref. [119]. Copyright 2021, American Chemical Society. **c-i** Cycling stability plot of discharge capacity over 200 cycles at 1 A g^−1^ for vacuum-annealed and flash-induced annealed Si anodes, with varying flash energy densities. **c-ii** Rate capability plot for Si anodes treated with FLA and FLF conditions. Reproduced with permission from Ref. [93]. Copyright 2021, American Chemical Society. **d-i** CV curves of a LITN-based capacitor obtained at scan rates ranging from 50 to 500 V s^−1^. **d-ii** Photograph of a packaged LITN capacitor unit alongside a commercially available AEC. **e-i** Schematic diagram of the assembled supercapacitor incorporating N-CNTs. Reproduced with permission from Ref. [222] Copyright 2023, Wiley–VCH. **e-ii** The charging–discharging curves of the N-CNT-based supercapacitor under different current densities. Reproduced with permission from Ref. [220] Copyright 2023, Wiley–VCH

Figure 15a-i features a photographic representation of a natural-stone-based MSC system consisting of a 3 × 3 array of electrochemical energy storage supercapacitors [98]. These supercapacitors were connected in series, demonstrating their ability to power LEDs. This system employed a capacitive Mn_3_O_4_ cathode, a faradaic Fe_3_O_4_ anode, and LiClO_4_ as the electrolyte salt material. A Cu current collector was intricately integrated onto a stone substrate through a laser-induced explosive reduction and sintering process initially applied to CuO NPs. The scan speed of a CW fiber laser (wavelength of 1070 nm) was modulated at a fixed power of 10 W to find the optimal condition for maximizing the conductivity and porosity of the Cu conductor, which are crucial for efficient electrochemical energy storage. In Fig. 15a-ii, a Ragone plot is presented to contextualize the performance of the laser-induced MSC stone cell. This plot compares the areal energy and power densities of the stone-based supercapacitor with those of previously reported MSC energy storage systems. The electrochemical stone module demonstrated outstanding energy density of 6.55 μWh cm^−2^ and a power density of 1.2 mW cm^−2^. A major advantage of this system is the recyclable nature of the stone substrate, which highlights its environmental sustainability and potential for circular economic applications.

Figure 15b-i depicts a flexible self-charging ceramic capacitor system with ultrafast and high-power density characteristics developed through an LLO transfer process [119]. This system combined a relaxor ferroelectric Pb(Mg_1/3_Nb_2/3_)O_3_–PbTiO_3_ (PMN–PT) capacitor and a piezoelectric PZT harvester, both of which were transferred from rigid substrates to a single PET film by optimizing the LLO conditions, including laser fluence, repetition rate, and scanning rate. The relaxor behavior in these ferroelectrics can be attributed to the disruption of long-range ferroelectric domain ordering and the formation of polar nanoregions, resulting in a relatively slim polarization–electric field (*P-E*) curve with a higher recoverable energy density and lower energy loss for capacitor applications. The flexible PMN-PT capacitor achieved a high recoverable energy density of 10.7 J cm^−3^ and an energy storage efficiency of 79.7% from P-E measurements at a maximum electric field of 600 kV cm^−1^. Concurrently, the PZT harvester of the self-charging capacitor system produced an open-circuit output voltage of 172 V and a short-circuit output current of 21 μA, triggered by biomechanical bending motions such as human finger movements. This generated energy was stored in the integrated, flexible capacitor, which could then be discharged with a high energy density of 2.58 J cm^−3^ within an ultrafast duration of 480 ns. This capability highlights the potential of the proposed approach for self-charging and energy storage to drive the development of flexible pulsed power electronic devices.

Photonic annealing has been used to modify LIB components under atmospheric conditions. Seok et al. reported enhanced performance of Si anodes in LIB systems through flashlight interactions under two distinct conditions: LILP, termed FLA, and HISP, referred to as FLF [93]. FLA process resulted in the inhomogeneous condensation of binders in the Si anode, whereas FLF method selectively promoted carbonization, oxidation, and pore formation on the surface of the Si anodes. Figure 15c-i presents cycling stability graphs that compare the as-dried, vacuum-annealed, and flash-induced samples to evaluate the effect of FLA treatment. The optimally flash-annealed sample exhibited superior cycling stability (FLA@0.77 J cm^−2^) of the Si anode, maintaining performance for up to 200 cycles at a specific current of 1 A g^−1^. This improvement was attributed to the partial flash-induced cross-linking between the polymer binders and Si particles. Figure 15c-ii illustrates the rate capability test results of the Si anode by varying the specific current from 0.5  to  5 A g^−1^ for FLA, FLF-1.3, and FLF-1.8 samples. The discharge capacities recorded during repeated cycles at a specific current of 2 A g^−1^ were 1010 mAh g^−1^ for FLA and 1710 mAh g^−1^ for appropriately conditioned (1.8 J cm^−2^) FLF, indicating that FLF significantly enhanced the rate capability of the Si anodes. The functionalized surfaces from the FLF provided efficient Li-ion and electron transfer largely because of their increased electrical conductivities, electrolyte affinity, and expanded pore structures.

Wang et al. reported a LITN nano-filament network that demonstrates high performance in MSCs [222]. Key processing parameters during the laser heating and transient cooling include the use of nanosecond pulse laser irradiation with a light intensity above 10^8^ W cm^−2^ and an energy density exceeding 10 J cm^−2^, which induce plasma formation and promote the diffusion and incorporation of nitrogen into molten titanium. The geometrically ordered nanoporous LITN structure has outstanding surface area, excellent chemical stability, and superior conductivity, enabling its use in high-frequency charging and discharging while enduring rapid volume changes during fast charge–discharge scenarios. Figure 15d-i shows the cyclic voltammetry (CV) curve of the LITN electrode at various voltage scanning speeds. The LITN electrode exhibited a quasi-rectangular CV curve with a slight redox peak at a scanning speed of 0.1 V s^−1^, indicating excellent rate performance and partial pseudo-capacitance behavior. As the scan rate increased to 10 V s^−1^ and above, the redox peaks gradually disappeared, demonstrating reduced interaction between electrolyte ions and the electrode while maintaining a complete CV curve shape at rates above 100 V s^−1^. They developed a surface-mountable LITN MSC, as shown in Fig. 15d-ii, which exhibits a long cycle life of 2 million cycles and an extraordinary volumetric energy density of 7.17 mWh cm^−3^ at 120 Hz in an aqueous electrolyte compared to conventional aluminum electrolytic capacitors (AECs). The LITN offers great potential for creating new materials and developing scalable integrated micro-devices with broad storage applications.

Zhang et al. [220] utilized millisecond-scale flash Joule heating to prepare N-CNTs for energy storage applications. Figure 15e-i shows the assembled supercapacitor device using the flash-prepared N-CNT samples as both anodic and cathodic electrodes with KOH electrolyte solution. Figure 15e-ii presents the galvanostatic charge–discharge curves of the capacitor device under different current densities ranging from 1 to 10 mA cm^−2^ with a voltage window of − 0.2 to 0.8 V. The area capacitance reaches 29.8 mF cm^−2^ at a current density of 1 mA cm^−2^ with an energy density of 1.03 µWh cm^−2^. All curves exhibit a triangular shape, even at the high current density of 10 mA cm^−2^, indicating ideal capacitive behavior. The charge–discharge cycle stability of the supercapacitor was investigated at a current density of 2 mA cm^−2^, showing an initial capacitance retention of 83% after 10,000 cycles. These results highlight the advantages of the flash heating method in preparing CNTs for advanced energy storage and conversion devices.

## Conclusions

**Fig. 16:**
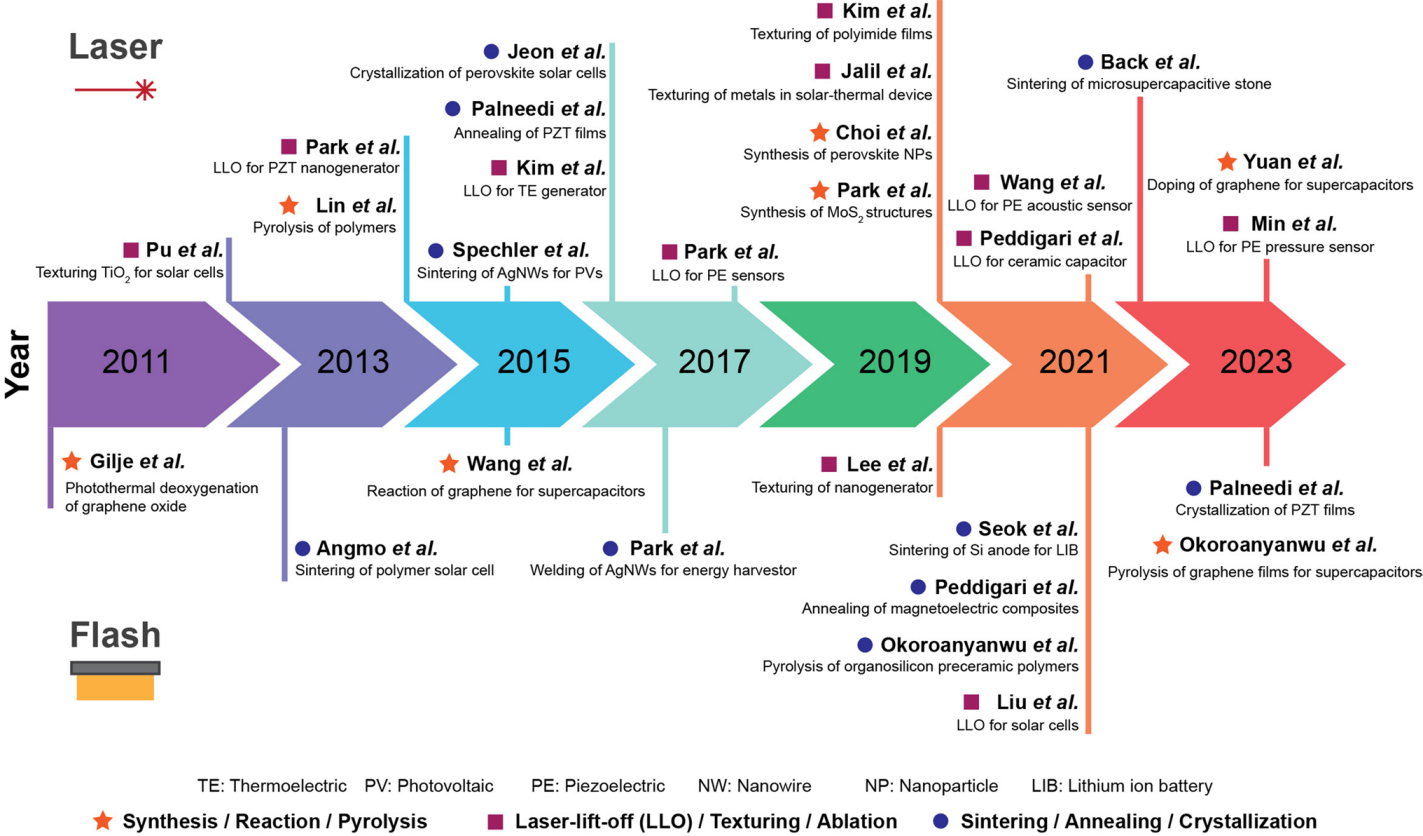
Comprehensive roadmap overview, which chronologically highlights the key developments in LMIs for energy conversion and storage applications

Major advances in LMI technology have contributed to robust solutions for energy conversion and storage applications, overcoming the limitations of traditional microfabrication and thermal processes. Laser and flash lamp light sources have been widely applied in numerous LMIs, including sintering, crystallization, lift-off, surface modification, carbonization, oxidation/reduction, doping, and synthesis, providing practical photothermal or photochemical strategies for numerous energy devices, ranging from batteries to self-powered electronics. Figure 16 presents a comprehensive roadmap that chronologically outlines and highlights the key developments in LMIs that are crucial for the progress of energy conversion and storage technologies.

However, there are several challenges in the commercialization of advanced energy materials and devices: (i) Extensive theoretical research on LMIs remains limited owing to their complex nature, characterized by factors such as nonequilibrium photon reactions, ultrafast interaction time, and LMI parameters with high degrees of freedom, which leads to multiple trials and errors in identifying optimized and consistent processing conditions. A vast database composed of multiscale simulations and feedback experimental results can be merged with machine learning and artificial intelligence to establish an efficient in-depth LMI model. (ii) The performance of light-induced energy devices should be improved to meet the increasing demand for higher efficiency in energy solutions. In this regard, synthesizing advanced materials with superior properties is crucial because they can be employed for novel LMIs, such as hierarchical sintering, large-scale crystal growth, and photochemical doping, to realize high-performance energy systems. (iii) Enhancing the spatial precision and controllability of LMI techniques is required for their high integration capability and uniformity, which enables precise design, fabrication, interconnection, and packaging technologies to demonstrate multifunctional and reliable energy applications. These challenges can be addressed by advancing optical technologies that can more accurately control the distribution of energy and time during LMIs. Enhancing uniformity in material synthesis and deposition is also required to improve the production yield of energy systems. (iv) Lastly, persistent progress is necessary in terms of improving mass production capability of intense LMI processes for cost-effective and sustainable manufacturing of energy applications. To address this issue, large-area processable light sources (e.g., line beam lasers, and flash lamps) along with optical beam shaping technologies can be introduced to enable required optical energy density over broad surfaces without sacrificing process quality and precision. We believe that a comprehensive review of key academic themes, significant efforts, and collaboration in the field of multidisciplinary research related to LMIs will promote future scientific innovations in energy materials and devices.

## Abbreviations

LMI: Light–material interaction
ESSs: Energy storage systems
IoTs: Internet of things
PZT: Pb(Zr_x_,Ti_1–x_)O_3_
PET: Polyethylene terephthalate
LLO: Laser lift-off
SEM: Scanning electron microscopy
f-TEG: Flexible thermoelectric generator
LMS: Laser multi-scanning
LED: Light-emitting diode
f-VLEDs: Flexible vertical light-emitting diodes
PLO: Photonic lift-off
PVs: Photovoltaic devices
PI: Polyimide
LAL: Light-absorbing layer
GSV: Granule spray in vacuum
CW: Continuous-wave
ITO: Indium tin oxide
XRD: X-ray diffraction
ME: Magnetoelectric
IPL: Intense pulsed-light
SSEs: Solid-state electrolytes
LLZO: Li_7_La_3_Zr_2_O_12_
SLS: Selective laser sintering
3D: Three-dimensional
LIBs: Li-ion batteries
R2R: Roll-to-roll
NCA: Nickel cobalt aluminum
PEN: Polyethylene naphthalate
DSC: Dye-sensitized solar cell
MSCs: Microsupercapacitors
ERS: Explosive reduction and sintering
LILP: Low-intensity and long-pulsed
HISP: High-intensity and short-pulsed
FLA: Flash light-induced annealing
FLF: Flash light-induced functionalization
μLEDs: Micro light-emitting diodes
NWs: Ag nanowires
CCs: Current collectors
TENGs: Triboelectric nanogenerators
PDMS: Polydimethylsiloxane
MMTENG: Magneto-mechano-triboelectric nanogenerator
GCCs: Graphene-copper composites
FPG: Flash-induced porous graphene
Vis–NIR: Visible–near-infrared
Ecs: Electrochemical capacitors
GO: Graphene oxide
LSG: Laser-scribed graphene
B-LIG-MSCs: Boron-doped laser-induced graphene microsupercapacitors
SSFL: Shaped femtosecond laser
NLSG: Nitrogen-doped laser-scribed graphene
rGO: Reduced graphene oxide
GF: Graphene liquid crystalline fibers
FH: Flash heating
NrGO: Nitrogen-doped reduced graphene oxide
FH-NrGO: Flash-heated nitrogen-doped reduced graphene oxide
2D: Two-dimensional
QDs: Quantum dots
FTS: Flash-thermal shock
NiS: Nickel sulfide
EDS: Energy-dispersive spectroscopy
N-CNTs: Nitrogen-doped carbon nanotubes
LCVP: Laser-driven chemical vapor pyrolysis
SiC: Silicon carbide
SiOC: Silicon oxycarbide
PCPs: Preceramic polymers
PLS: Print light synthesis
TSP: Touchscreen panel
f-μLEDs: Flexible micro-light-emitting diodes
AD: Aerosol-deposited
WPBPS: Wearable piezoelectric blood pressure sensor
DBP: Diastolic blood pressure
FDA: Food and Drug Administration
iPANS: Inorganic PZT acoustic nanosensor
PMN-PZT: Pb(Mg_1/3_Nb_2/3_)O_3_–Pb(Zr,Ti)O_3_
PMN–PT: Pb(Mg_1/3_Nb_2/3_)O_3_–PbTiO_3_
CV: Cyclic voltammetry
